# Development and Characterization of MYB-NFIB Fusion Expression in Adenoid Cystic Carcinoma

**DOI:** 10.3390/cancers14092263

**Published:** 2022-04-30

**Authors:** Joseph O. Humtsoe, Hyun-Su Kim, Leilani Jones, James Cevallos, Philippe Boileau, Fengshen Kuo, Luc G. T. Morris, Patrick Ha

**Affiliations:** 1 Department of Otolaryngology, Head and Neck Surgery, University of California-San Francisco, Helen Diller Family Comprehensive Cancer Center, San Francisco, CA 94080, USA; joseph.humtsoe@ucsf.edu (J.O.H.); corkim@ucdavis.edu (H.-S.K.); leilani.jones@ucsf.edu (L.J.); 2 School of Medicine, University of California-San Francisco, San Francisco, CA 94080, USA; james.cevallos@ucsf.edu; 3 Graduate Group in Biostatistics, Center for Computational Biology, University of California-Berkeley, Berkeley, CA 94720, USA; philippe_boileau@berkeley.edu; 4 Department of Surgery, Memorial Sloan Kettering Cancer Center, New York, NY 10021, USA; kuof@mskcc.org (F.K.); morrisl@mskcc.org (L.G.T.M.)

**Keywords:** adenoid cystic carcinoma, chromosomal t(6;9) translocation, MYB, MYB-NFIB fusion

## Abstract

**Simple Summary:**

Chromosomal t(6;9) translocation is commonly found in adenoid cystic carcinoma (ACC) of the salivary gland. This genetic rearrangement results in the fusion of MYB and NFIB genes. Despite the frequent occurrence of t(6;9) translocation and MYB-NFIB gene fusion, the nature of chimeric MYB-NFIB proteins and their potential relevance in the development and behavior of ACC remains poorly understood. The lack of validated ACC cell lines, harboring the t(6;9) translocation with defined MYB-NFIB fusion protein, has restricted fusion-specific functional studies. Hence, we sought to characterize and establish in vitro and in vivo models of a MYB-NFIB fusion protein expressing system in ACC. Defining the nature and functional aspect of MYB-NFIB fusion proteins may not only improve our understanding of the disease but also contribute to the identification of molecular targets that are druggable and can be developed for future therapeutic purpose.

**Abstract:**

Adenoid cystic carcinoma (ACC) is the second most common cancer type arising from the salivary gland. The frequent occurrence of chromosome t(6;9) translocation leading to the fusion of MYB and NFIB transcription factor genes is considered a genetic hallmark of ACC. This inter-chromosomal rearrangement may encode multiple variants of functional MYB-NFIB fusion in ACC. However, the lack of an ACC model that harbors the t(6;9) translocation has limited studies on defining the potential function and implication of chimeric MYB-NFIB protein in ACC. This report aims to establish a MYB-NFIB fusion protein expressing system in ACC cells for in vitro and in vivo studies. RNA-seq data from MYB-NFIB translocation positive ACC patients’ tumors and MYB-NFIB fusion transcript in ACC patient-derived xenografts (ACCX) was analyzed to identify MYB breakpoints and their frequency of occurrence. Based on the MYB breakpoint identified, variants of MYB-NFIB fusion expression system were developed in a MYB-NFIB deficient ACC cell lines. Analysis confirmed MYB-NFIB fusion protein expression in ACC cells and ACCXs. Furthermore, recombinant MYB-NFIB fusion displayed sustained protein stability and impacted transcriptional activities of interferon-associated genes set as compared to a wild type MYB. In vivo tumor formation analysis indicated the capacity of MYB-NFIB fusion cells to grow as implanted tumors, although there were no fusion-mediated growth advantages. This expression system may be useful not only in studies to determine the functional aspects of MYB-NFIB fusion but also in evaluating effective drug response in vitro and in vivo settings.

## 1. Introduction

Adenoid cystic carcinoma (ACC) is a common malignancy of salivary glands [1,2]. Generally characterized as a slow growing tumor, ACC has the propensity to recur and develop into a late distant metastasis [3,4,5]. Surgical intervention with or without radiation therapy remains the primary modality, but unfortunately, patients with recurrent and unresectable ACC are often untreatable due to their limited response to systemic therapies [6]. Currently, there are no approved drugs and for optional treatment, a clinical guideline developed for managing salivary gland malignancy, recommend the use of multitargeted tyrosine kinase inhibitors, such as lenvatinib or sorafenib for patients with ACC [7]. Several studies are ongoing to identify therapeutic targets and develop effective drugs to benefit ACC patients [6,8].

Chromosomal translocations are commonly found in many types of hematologic and solid cancers [9]. They contribute to oncogenesis as a consequence of promoter and enhancer element rearrangements that lead to dysregulated expression of critical genes, or as a result of chimeric gene products that arise from gene fusions with kinase or transcription factor activities [10,11]. Abnormal chromosomal arrangements are also commonly found in salivary gland malignancy [12]. In ACC, the most frequently identified is the chromosomal t(6;9) translocation [12,13]. The chromosome exchange results in the fusion of the transcription factor genes, MYB and NFIB, and is considered a hallmark of ACC [14,15]. In addition to the abnormally high MYB expression due to promoter/enhancer element exchanges [16], the inter-chromosomal rearrangement produces a MYB-NFIB gene fusion transcript that may encode chimeric fusion protein. Depending on the breakpoint location that links the two genetic elements, multiple variants of MYB-NFIB gene fusion transcripts can be generated [17].

Despite their frequent occurrence with functional potential that may be crucial in the progression and behavior of ACC, the activities of chimeric MYB-NFIB fusion proteins remain largely unknown. A number of established ACC patient-derived-xenografts (ACCX) are readily available [18,19], however, the lack of validated ACC cell lines, harboring the t(6;9) translocation, has limited studies to unravel the functional aspect of MYB-NFIB fusion in ACC.

Here, we describe the development of an inducible MYB-NFIB fusion model based on MYB breakpoint sites identified from t(6;9) translocation positive patient-derived xenograft and patients’ tumors of ACC. This expression system may be useful not only in studies to understand the role of MYB-NFIB fusion but also help in evaluating approaches to test effective drug response in vitro and in vivo models.

## 2. Materials and Methods

### 2.1. ACC Patient-Derived Xenografts and Cell Lines

Patient-derived xenografts of ACC (ACCX2, ACCX6, ACCX5M1, ACCX9, ACCX11, ACCX20M1, and ACCX38M1) obtained from Dr. C.A. Moskaluk (University of Virginia, Charlottesville, VA, USA) were passaged in mice as previously described [20]. MDA-ACC-01 (ACC-01) and UM-HACC-2A (HACC-2A) cell lines derived from salivary gland ACC were provided by Dr. Adel El-Naggar (UT MD Anderson Cancer Center, Houston, TX [21], and Dr. Jacques Nor (University of Michigan, Ann Arbor, MI, USA) [22], respectively. Cell culture media and conditions were followed as previously described [20]. Cell lines were authenticated by STR-profiling at the Bioscience Facility, University of California, Berkeley, CA, USA.

### 2.2. Patient Samples and MYB Breakpoint Identification

ACC patient samples and RNA sequencing (RNA-Seq) have been described in our previous report [23]. In this study, RNA-seq data from 10 ACC patient tumors that were identified to harbor the t(6;9) translocation were retrieved for further evaluation.

To identify MYB breakpoints from the bulk of RNA-seq data, we combined NeoFuse pipeline (version 1.1.1) and Arriba command-line analysis tools [24,25]. Briefly, FASTQ files of RNA-seq reads were subjected to NeoFuse, which employed STAR for aligning RNA-seq reads against human hg19 (GRCh37.p13) genome along with GENCODE gene model (v19) for calling fusions. Once read alignment were complete, Arriba was used in the NeoFuse pipeline for building the list of fusion calls. Default pipeline parameters used in the analysis allowed us to acquire the final fusions list with gene annotation and break point information after QC filtering.

### 2.3. MYB-NFIB Expression Analysis, Cloning and Generation of MYB-NFIB Fusion Constructs

Total RNA extracted from ACCXs was used for reverse transcription into cDNA using Superscript First-Strand Synthesis kit (Invitrogen, ThermoFisher Scientific, Waltham, MA, USA). To confirm detection of wild type MYB (MYB^wt^) and MYB-NFIB expression in ACCXs, PCR analysis was performed with Taq polymerase (Qiagen, Germantown, MD, USA) using Mex6F (MYB exon 6 forward), and Mex9R (MYB exon 9 reverse) primers, or with Mex6F and Nex11R (NFIB exon 11 reverse) primers set. For positive reference, we used plasmid HA-tagged MYB (MYB^wt^ variant 2) and HA-MYB-NFIB (MYB exon 8-NFIB exon 10 fusion) constructs [26].

For identification of MYB-NFIB transcript, reverse transcribed cDNA from ACCX11, ACCX29 and ACCX20M1 was used. Full-length fusion transcripts were amplified using Platinum^TM^ PCR Supermix High-Fidelity (Invitrogen) with MYB-orf-F (located upstream of MYB start codon) and NFIB-orf-R (located downstream of NFIB stop codon) primers set. PCR-amplified bands were gel-extracted, purified, and analyzed by Sanger sequencing (Quintara Biosciences, Hayward, CA, USA). Sequence-verified PCR-product or HA-tagged MYB^wt^ plasmid cDNA were then used as templates for second-round PCR for cloning MYB-NFIB fusion or MYB^wt^ into Gateway pDONR vector using attMYB and attNFIB primers. Following transformation, individual positive pEntry clones (pDONR with inserted PCR fragment) were selected and re-verified by sequencing.

Next, pEntry constructs with respective MYB-NFIB and MYB^wt^ inserts were recombined into a pLVX-TetOn-hygro-C-3xFLAG expression vector using Gateway cloning system (Gateway BP/LR Clonase II, Invitrogen). The various constructs were further verified by sequencing. FLAG-tagged MYB^wt^ or MYB-NFIB constructs along with viral elements (VSV-G and pRH gag, pol) were transfected into HEK293T cells to generate lentiviral particles. Virus-transduced ACC stable cell lines were generated after selection in hygromycin (200 μg/mL). To induce expression of FLAG-tagged MYB^wt^ or MYB-NFIB constructs, stable ACC-01 and HACC-2A cells were cultured in media containing doxycycline (0.5 μg/mL). All Gateway cloning lentiviral vectors were obtained from Dr. Nevan Krogan’s Lab (Gladstone Institute, UCSF, San Francisco, CA, USA). Detailed primer information is provided in Table S1.

### 2.4. Biochemical Analysis

Protein extracts prepared from fresh or frozen ACCX tissues or cell cultures were resolved in SDS-polyacrylamide gel and analyzed by immunoblotting [20]. MYB^wt^ or MYB-NFIB fusion protein were detected with anti-MYB (N-terminus epitope) D2R4Y, cat #12319 (Cell Signaling Tech. Inc, Danvers, MA, USA); anti-MYB (N-teminus epitope) EP769Y, Ab45150 (Abcam., Waltham, MA, USA), anti-MYB (C-terminus epitope), D-7, Sc-74512 (Santa Cruz Biotech. Inc., Dallas, TX, USA), or anti-FLAG, M2 (Sigma-Aldrich, Inc., St. Louis, MO, USA). For protein stability assay, time chase experiments were performed in cells expressing MYB^wt^ or MYB-NFIB fusion. Briefly, cells were cultured in doxycycline (dox) containing media for 24 h to induce the expression of MYB^wt^ or MYB-NFIB fusion. Cells were then refreshed with media without dox for up to 6 hr. Alternatively, in cycloheximide (CHX) chase experiment, dox-treated cells were incubated in media containing CHX (100 μg/mL) to prevent synthesis of new protein. Lysates were then prepared and analyzed by immunoblotting followed by densitometry to determine half-life of MYB^wt^ and MYB-NFIB fusion protein.

### 2.5. RNA Library Generation, Sequencing and Alignment

Total RNA was extracted from four replicates each of doxycycline-induced MYB^wt^, M8:N10 (MYB-NFIB) and empty vector-transduced ACC-01 cells (control). RNA sample integrity was assessed with an Agilent Bioanalyzer, and corresponding RNA libraries were constructed using TruSeq strand mRNA Library Prep kit (Illumina). The RNA libraries were sequenced using the Illumina HiSeq4000 system with 50 base paired end reads (Functional Genomic Core Facilities, UCSF). Sample sequences were aligned to the Ensembl Human GRCh38.78 reference genome using STAR [27] after sample quality checks.

### 2.6. RNA-Seq Analysis

The count matrix produced by STAR was used for the RNA-seq analysis. Low abundance genes, defined as having fewer than 10 counts in less than three samples, were excluded. Pairwise differential gene expression analyses of sample types were then performed using the DESeq2 [28] R/Bioconductor package [29]. Effect size estimates were corrected using the apeglm method by Zhu et al. [30]. A false discovery rate (FDR) cutoff of 0.05 was used to define differentially expressed genes. To identify pathway enriched terms, Reactome pathway enrichment analyses [31] were performed using the ReactomePA R package [32].

### 2.7. Gene Expression Validation by RT-qPCR

Total RNA purified from dox-induced cell lines or ACCX were reverse transcribed to cDNA with iScript Reverse Transcription mix and amplified with specific primers (Table S1) using iTag Universal SYBR Green (Bio-Rad, Inc., Hercules, CA, USA). The fold change or relative gene expression was determined with TBP gene as internal control using the 2^−^^ΔCt^ or 2^−^^ΔΔCt^ equation, respectively.

### 2.8. In Vivo Xenografts Assay

Parental ACC-01 cells, or cells expressing either FLAG-tagged MYB^wt^ or MYB-NFIB fusion constructs, were implanted subcutaneously into the flanks of NOD.Cg-*Prkdc*^scid^*Il2rg^tm1Wjl^*/SzJ mice (The Jackson Laboratory, Bar Harbor, ME, USA). Mice were fed with doxycycline (0.5 mg/mL doxycycline and 5% sucrose) containing water, and tumor growth activity was monitored. After 2.5–3 months, mice were sacrificed, and tumors were harvested for further analysis. Mice were housed and cared in animal facilities as approved by the UCSF Animal Care and Use Committee.

### 2.9. Additional Statistical Analysis

Data presented are representative of replicate experiments wherever indicated. Graphical representations were performed in GraphPad Prism v/9.

## 3. Results

### 3.1. Heterogenous MYB Breakpoint Loci in MYB-NFIB Fusion Positive ACC

**Figure 1:**
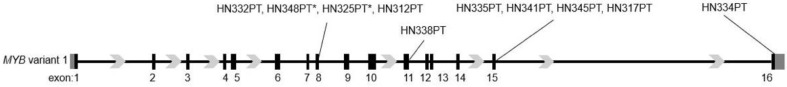
Schematic of MYB locus showing breakpoint locations. RNA-seq data from the indicated cases of ACC patient tumors was analyzed by combined NeoFuse and Arriba tool to identify MYB breakpoints. * Indicates cases where MYB breakpoint was also detected at exon 7.

In a previous study, we found that 44% of tumor samples from ACC patients harbored the MYB-NFIB gene translocation [23]. To identify the MYB-NFIB fusion variants present in these cases, we examined the RNA-seq data from MYB-NFIB translocation positive samples. The Arriba tool-processed fusion data output revealed that MYB breakpoint sites were variable that occurred from exon 7 through exon 16 (Figure 1 and Table S2).

Of the ten tumors data analyzed, MYB breakpoint occurred at exon 8 for tumors HN332PT, HN348PT, HN325PT and HN312PT, and at exon 15 for tumor samples HN335PT, HN341PT, HN345PT, and HN317PT. A MYB breakpoint at exon 11, and another at exon 16 were detected in tumor samples HN338PT and HN344PT, respectively. Analysis also revealed that all four cases detected with MYB breakpoints at exon 8 contained closely related variants with breakpoints located at nine nucleotides upstream of the C-terminal end of exon 8. Furthermore, tumors HN348PT and HN325PT also showed the presence of an additional variant with a MYB breakpoint occurring at exon 7. These findings indicate that MYB breakpoint occurs predominantly downstream of exon 8. Although not determined in this study, NFIB breakpoint sites linking to MYB detected through Arriba analysis occurred mostly with the last two exons (Table S2).

### 3.2. MYB-NFIB Fusion Expression in ACC Patient-Derived-Xenograft (ACCX)

**Figure 2:**
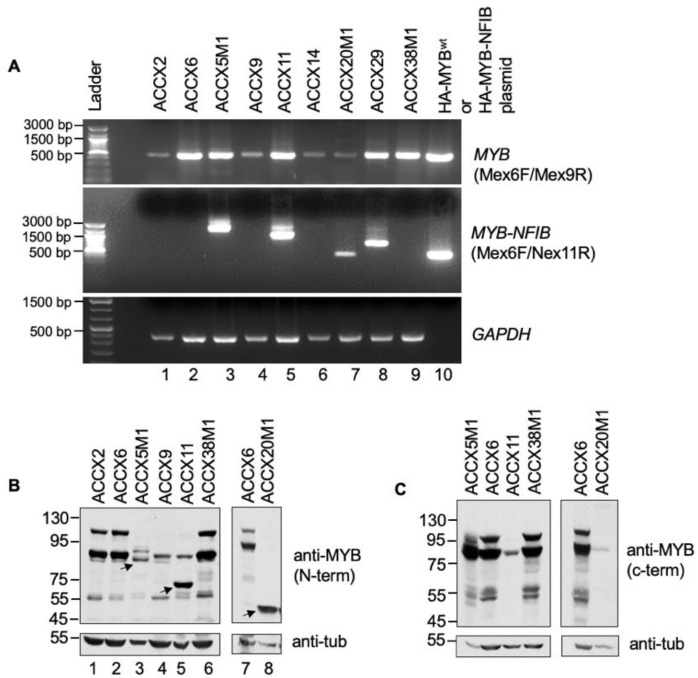
Expression of MYB and MYB-NFIB fusion in ACCXs. (**A**). RNA extracted from the indicated ACCXs were evaluated for expression of MYB-NFIB fusion or MYB^wt^ by RT-PCR. HA-tagged MYB^wt^ or HA-MYB-NFIB plasmid cDNA were included for reference control. GAPDH was used as input control. (**B**). Protein lysates from ACCXs were analyzed using an antibody that recognizes an N-terminal epitope of MYB. Arrows indicate smaller MYB bands. They correlate with the presence of MYB-NFIB product detected in “A” and likely represent MYB-NFIB fusion protein. (**C**). Protein lysates as in “B” were analyzed using an antibody that recognizes a C-terminal epitope of MYB. Note that the smaller MYB band from “B” is undetectable.

To examine the expression of MYB-NFIB fusion in ACC patient-derived xenografts (ACCXs), we performed a semi-quantitative RT-PCR analysis of RNA extracted from a panel of nine ACCXs that are known to harbor or lack the MYB-NFIB translocation. Initially, a forward primer Mex6F (MYB exon 6) with either Nex11R (reverse primer from NFIB exon 11) or Mex9R (MYB exon 9) were used for the analysis. Plasmid cDNA of HA-tagged wild type MYB (MYB^wt^) and MYB-NFIB fusion (C8:N10) constructs were included for reference controls [26]. Result shows that PCR products of varying sizes were detected in ACCX5M1, ACCX11, ACCX14, ACCX20M1 and ACCX29 (Figure 2A).

The results correspond with the MYB-NFIB translocation status described for these ACCXs [18], and indicates that the different band sizes may represent MYB-NFIB fusion variants with different MYB breakpoint sites. Although MYB breakpoints in ACCX5M1, ACCX11, and ACCX14 has been described [33], the breakpoint locations and its’ fusion with NFIB region in ACCX29 and ACCX20M1 are not clear. The product size from ACCX20M1 is identical with plasmid fusion construct (Figure 2A, lane 7 and 10) and may represent a fusion with a MYB breakpoint at exon 8. The band size in ACCX29 also reflected the presence of a MYB-NFIB fusion but with a distinct MYB breakpoint site. All ACCX samples that do not harbor MYB-NFIB translocation were positive for MYB^wt^, albeit with variable expression levels.

Next, we evaluated MYB^wt^ and MYB-NFIB fusion protein expression status in ACCXs by Western blotting. ACCX14 and ACCX29 tissue samples for protein lysate were not accessible and was excluded in this analysis. Our results show that a N-terminus epitope MYB antibody detected two major bands in ACCX2, ACCX6 and ACCX38M1 (Figure 2B and Figure S1). In comparison, relatively lower levels of the two bands were detected in ACCX5M1, ACCX9, ACCX11 and ACCX20M1. Interestingly, smaller but distinct MYB protein bands were detected in ACCX5M1, ACCX11 and ACCX20M1 (Figure 2B lane 3, 5 and 8). Although expression of splice variants of MYB are possible, it is likely that these smaller bands represent MYB-NFIB fusion proteins. Since MYB-NFIB fusion lacks the carboxyl-terminus domain of MYB, we analyzed the lysates with a C-terminus epitope MYB antibody. The results show that while the two major bands were detected in ACCX6 and ACCX38M1, the smaller protein bands were undetectable in ACCX5M1, ACCX11 and ACCX20M1 (Figure 2C and Figure S1). Together, these results not only confirm the presence of MYB-NFIB fusion gene product but also show the expression of a cyto-domain lacking MYB protein at a detectable level that likely represent MYB-NFIB fusions proteins in selected ACCXs.

### 3.3. Identification and Generation of MYB-NFIB Fusion from ACCXs

**Figure 3:**
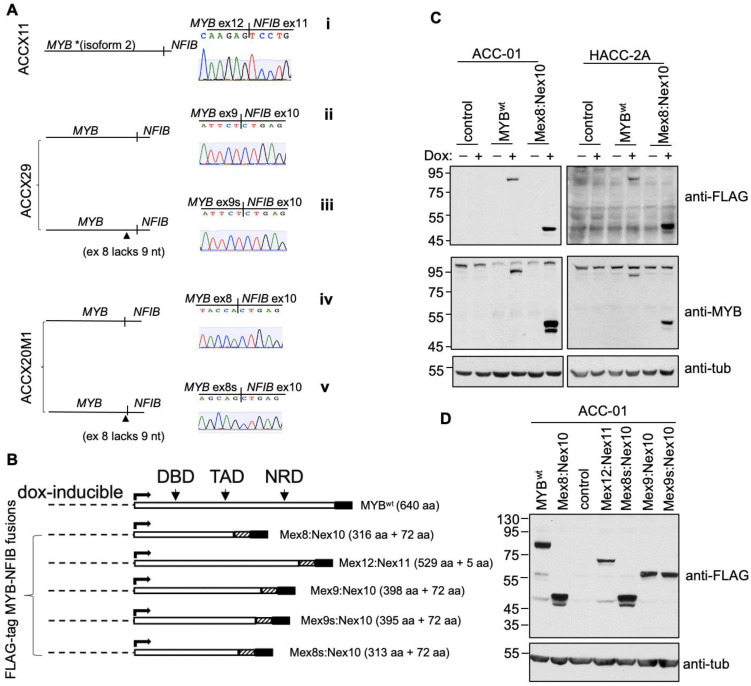
Expression and characterization of MYB-NFIB fusion. (**A**), Sequencing chromatogram showing MYB-NFIB fusion junctions as derived from three ACCXs. Two variants of MYB-NFIB fusion were detected in ACCX29 (ii and iii) and ACCX20M1 (iv and v) with its second form (iii and v) representing a variant lacking 9 nucleotides on MYB exon 8. * Note that in MYB-NFIB fusion from ACCX11 (i), MYB region represent isoform 2 as sequencing revealed the lack of exon 10 normally present in the larger isoform 1. (**B**), Schematic representation of dox-inducible FLAG-tagged MYB^wt^ and MYB-NFIB fusion constructs. Striped and shaded box represents the position of NFIB and FLAG-tag, respectively. DBD: DNA binding domain; TAD transactivation domain; NRD: negative regulatory domain. (**C**), Immunoblotting analysis of MYB^wt^ and MYB-NFIB fusion expression in ACC-01 and HACC-2A cells following dox induction. Complementary antibodies (anti-FLAG and N-terminal epitope anti-MYB) were used to verify the expression. (**D**), ACC-01 cells expressing the various MYB-NFIB fusion variants were verified by immunoblotting with anti-FLAG. In A, B and D, the ”s” represent the absence of 9 nucleotides.

Next, the full length MYB-NFIB fusion gene products expressed in ACCX11, ACCX29 and ACCX20M1 were PCR-amplified, sub-cloned and sequenced. The analysis confirmed the expression of bona fide MYB-NFIB fusion transcripts (Figure 3A, i–iv). In ACCX11, the fusion gene was composed of MYB exon 12 (MYB isoform 2) spliced to exon 11 of NFIB. In ACCX29, the fusion was between MYB exon 9 and NFIB exon 10, while in ACCX20M1, the fusion occurred between MYB exon 8 and exon 10 of NFIB. As predicted, MYB-NFIB fusion from ACCX20M1 was identical to the reference plasmid MYB-NFIB-fusion construct used in this study. In both ACCX29 and ACCX20M1, we confirmed the presence of a closely related second variant that lacked nine in-frame nucleotides in exon 8 of MYB (Figure 3A, iii and v).

The various MYB-NFIB fusion transcripts or the wild type MYB (MYB^wt^) were then cloned into a dox-inducible FLAG-tagged expression system (Figure 3B). Based on the position of the MYB exon site linking to NFIB, the FLAG-tagged MYB-NFIB fusion constructs were also referred to individually as Mex8:Nex10, Mex8s:Nex10, Mex12:Nex11, Mex9:Nex10 and Mex9s:Mex10. The various constructs were then stably introduced into ACC-01 or HACC-2A cells to express either MYB-NFIB fusion or MYB^wt^ following dox-induction. ACC-01 and HACC-2A are validated ACC cell lines derived from ACC patient tumors with an identified t(6;14) and t(6;9) translocation, respectively [21,22]. Notably, ACC-01 was found to lose the translocation over passages. While the presence of other MYB types (MYBL1 and MYBL2) remain to be characterized, both of these cell lines are known to express relatively low levels of MYB^wt^. More importantly, ACC-01 cells are MYB-NFIB fusion negative and with HACC-2A, the fusion can only be verified using nested-PCR techniques, such that on Western blot, we could not identify any appreciable MYB-NFIB fusion protein. Thus, we utilized these two authenticated ACC cell lines for ectopic MYB-NFIB fusions expression studies. Results show that MYB^wt^ and MYB-NFIB fusion proteins were efficiently expressed following dox induction as detected with either anti-FLAG or N-terminus epitope MYB antibody (Figure 3C and Figure S2). Similarly, dox-induced expression of Mex12:Nex11, Mex9:Nex10, Mex9s:Mex10, and Mex8s:Nex10 fusion constructs were confirmed by FLAG antibody in ACC-01 cells (Figure 3D and Figure S2).

Furthermore, we confirmed that the smaller MYB protein band detected in ACCX11 and ACCX20M1 corresponded to fusion constructs Mex12:Nex11 and Mex8:Nex10, respectively (Figure S3). Currently, there are no specific antibodies available that selectively detect MYB-NFIB fusion protein. Since Mex12:Nex11 and Mex8:Nex10 constructs were derived from ACCX11 and ACCX20M1, respectively, these results provide evidence that the two ACCXs express MYB-NFIB fusion protein at a detectable level.

Next, we assessed the impact of MYB-fusion expression in cell growth. Results showed that ACC-01 and HACC-2A cell growth activities in the absence or presence of dox-induction remained comparable (Figure S4). This data indicated that expression of MYB-fusion or MYB^wt^ does not enhance cell growth activities in the two-cell line model.

### 3.4. Increased Stability of MYB-NFIB Fusion in ACC Cells

**Figure 4:**
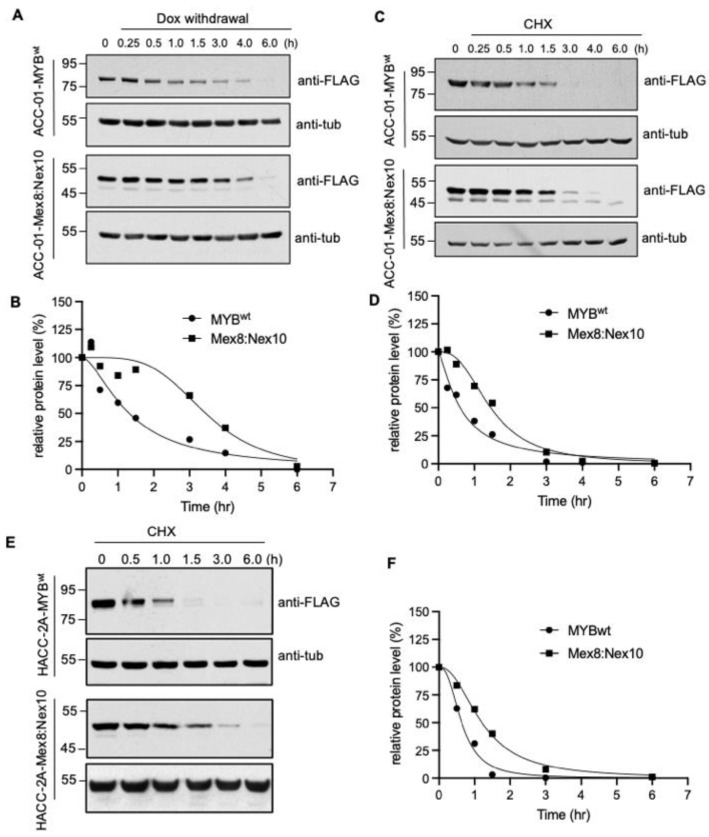
Differential protein stability of MYB^wt^ and MYB-NFIB fusion. (**A**,**B**), Stable ACC-01 cells harboring FLAG-tagged MYB^wt^ or MYB-NFIB fusion were dox-induced for 24 h, then re-cultured in media without dox for the indicated time period. Cell lysates were prepared and analyzed by Western blotting followed by densitometry to determine half-life of MYB^wt^ and MYB-NFIB fusion. (**C**,**D**), Cells as above induced with dox for 24 h were treated with cycloheximide (CHX; 100 ug/mL) for the indicated time period. Cell lysates were prepared and subjected to immunoblotting and densitometric analysis. (**E**,**F**), HACC-2A cells expressing dox-induced FLAG-tagged MYB^wt^ or MYB-NFIB fusion were incubated with CHX and analyzed as above.

To determine the stability of MYB-NFIB fusion, which lacks C-terminal domain of MYB^wt^, cells expressing the Mex8:Nex10 or MYB^wt^ constructs were analyzed after withdrawal of dox in culture media or treatment with cycloheximide. Withdrawal of dox ceases the expression of fresh inducible protein, while cycloheximide inhibits new protein synthesis and thereby monitoring the MYB^wt^ and MYB-NFIB protein level over a time period allowed us to determine their degradation rate. Results from dox withdrawal assay showed that the half-life of MYB^wt^ was about 90 min, while MYB-NFIB fusion exhibited a half-life period of about 2.6 fold higher than MYB^wt^ (Figure 4A,B and Figure S5).

Cycloheximide chase experiment also showed a rapid protein turnover of MYB^wt^ relative to MYB-NFIB fusion, which displayed a half-life of about 2.4 fold higher than that of MYB^wt^ (Figure 4C,D and Figure S5). Furthermore, a comparable pattern of MYB^wt^ and MYB-NFIB fusion protein degradation was found in HACC-2A cells (Figure 4E,F and Figure S5). Results from additional fusion constructs also showed that MYB-NFIB protein demonstrated a delayed degradation pattern as compared to MYB^wt^ (Figure S6). Together, these results demonstrated that MYB-NFIB fusion is more stable and is less vulnerable to rapid protein turnover compared to its parent MYB^wt^.

### 3.5. MYB-NFIB Fusion Expression Enriches Genes Associated in Interferon Signaling Pathway

**Figure 5:**
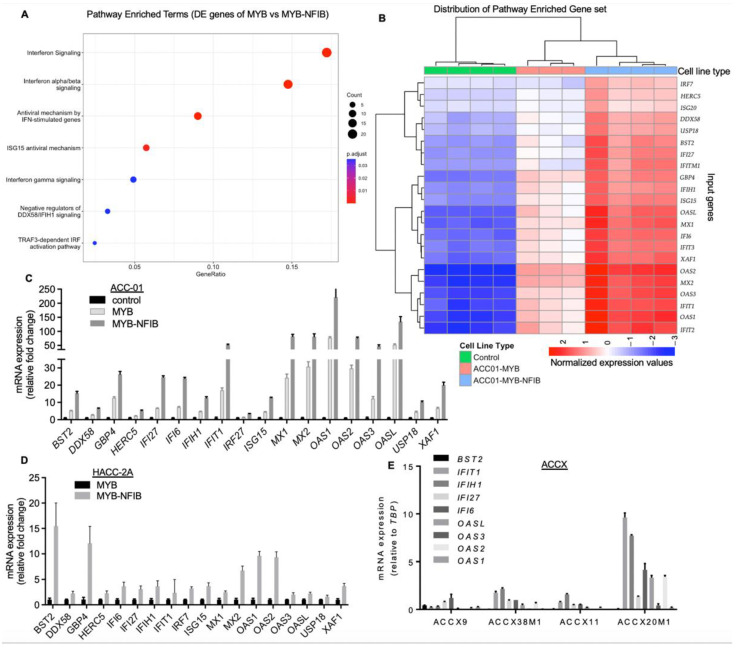
Correlation of MYB-NFIB fusion expression with gene enrichment associated in interferon (IFN) signaling pathway. (**A**), Reactome pathway enrichment analysis of 215 annotated genes (FDR < 0.05) found in the MYB^wt^ versus MYB-NFIB fusion differential gene expression analysis. The x-axis indicates the proportion of genes from this set contained in each pathway’s gene set. Twenty-two IFN pathway-associated genes were found in all. (**B**), Heat-map representation of the normalized and centered gene expression data of the 22 IFN signaling pathway-associated genes. Samples and genes were ordered using the hierarchical clustering with complete linkage of their Euclidean distances. Note: one MYB^wt^ sample was excluded from the overall analysis because its RNA sequencing read counts were too low to be included in the HiSeq4000 input. (**C**), Validation of the upregulated genes by qPCR. RNA extracted from parental control, MYB^wt^ or MYB-NFIB fusion cells incubated in dox-media for 24 h were analyzed for the expression of the various gene targets. (**D**), qPCR analysis of the target genes in HACC-2A cells expressing MYB^wt^ or MYB-NFIB fusion. (**E**), RNA extracted from the indicated ACCXs were evaluated for the expression of representative gene targets as shown.

To evaluate whether MYB-NFIB fusion acquired altered transcriptional activities distinct from wild type MYB, gene expression profiles of MYB^wt^, MYB-NFIB and ACC-01 parental control cells were analyzed by RNA-sequencing. For this study, we selected the M8:N10 construct as the representative MYB-NFIB fusion. A total of 16,118 genes were considered in the differential expression analyses. After adjusting for multiple tests (FDR < 0.05), 1815 genes were found to be significantly differentially expressed between the MYB-NFIB and MYB^wt^ samples. We filtered this set of differentially expressed genes further by applying a fold-change cutoff of two, using MYB^wt^ samples as reference, and retained 215 genes after removing unannotated genes (Table S3). A Reactome pathway enrichment analysis of these genes revealed a strong association with interferon (IFN) signaling pathways (Figure 5A).

We acquired all genes featured in the enriched pathways along with corresponding genes from the control set and performed a hierarchical clustering. This allowed us to compare the unique gene set expression profile across the controls, MYB^wt^ and MYB-NFIB fusion groups. Notably, IFN signaling pathway-associated genes were distinctly upregulated in MYB-NFIB fusion compared to MYB^wt^ samples, with the latter being higher relative to the control group (Figure 5B). Genes identified in these specific pathways were IFN inducible factors such as, IFIT1, IFIT2, IFIT3, IFITM1, MX1, MX2, BST2, ISG15, ISG20, as well as members of the 2′-5′-Oligoadenylate Synthetase, comprising of OAS1, OAS2, OAS3 and OASL [34,35]. Others include genes related to IFN pathways like, DDX58, USP18, GBP4, IRF7, and XAF1. Quantitative PCR analysis validated the elevated expression of these gene sets in MYB-NFIB fusion cells relative to MYB^wt^ or the control ACC-01 cells (Figure 5C). Similar analysis in HACC-2A cells confirmed that the IFN signaling pathway-associated genes were upregulated in MYB-NFIB fusion expressing cells compared to the MYB^wt^ cells (Figure 5D).

Next, we compared the expression status of IFN pathway genes in four representative ACCXs that either lack (ACCX9, ACCX38M1) or harbor (ACCX11 and ACCX20M1) the MYB-NFIB fusion. Quantitative PCR results show that a number of the target genes detected appear modest and at a comparable level across ACCX9, ACCX38M1 and ACCX11, while a relatively higher expression pattern of several genes were observed in ACCX20M1 (Figure 5E). Although, only one fusion-positive PDX correlated with the gene upregulation, these results overall, indicate that MYB-NFIB fusion activity may be associated in the enrichment of genes sets involved in the interferon-related signaling pathway.

### 3.6. Development of MYB-NFIB Fusion Expressing ACC Cells for In Vivo Model

**Figure 6:**
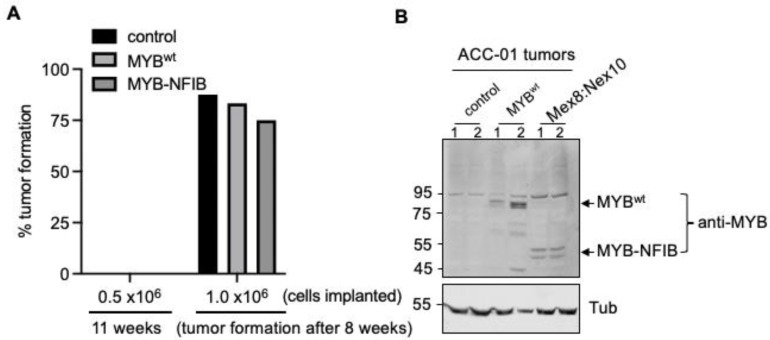
In vivo tumor growth analysis of MYB-NFIB fusion expressing ACC-01 cells. (**A**), Mice were implanted subcutaneously with parental or stable cell lines of FLAG-tagged MYB^wt^ or MYB-NFIB fusion constructs as indicated (*n* = 6–8 each group). Mice were fed with dox containing drinking water. Tumor growth was then assessed up to 11 weeks. Palpable development of tumor at the end of 8 weeks was considered positive tumor growth. (**B**), Protein extracts from two representative tumors of each group were analyzed for MYB^wt^ or MYB-NFIB fusion expression by immunoblotting.

Finally, we sought to evaluate whether MYB-NFIB fusion expression is permissive to form and support tumor growth in vivo. To test this, tumor forming capacity by MYB-NFIB fusion (Mex8:Nex10) was compared with MYB^wt^ and parental ACC-01 cells in two mice groups implanted with either 0.5 × 10^6^ or with 1.0 × 10^6^ cells. Data showed that in the mice group implanted with 0.5 × 10^6^ cells, no tumors were formed either with the MYB-NFIB fusion, MYB^wt^ or parental control cells during an 11-week monitoring time period (Figure 6A).

In mice implanted with 1.0 × 10^6^ cells, palpable tumors were formed in parental (3/8), MYB-NFIB fusion (1/6) and MYB^wt^ (1/6) at about 5.2 weeks. And by the end of 8 weeks, tumor formation occurred with parental control (87.5 %), MYB^wt^ (83.3%) and MYB-NFIB fusion (75%) cells. Evaluation of the tumors by Western blotting verified the positive expression of the dox-induced MYB-NFIB fusion and MYB^wt^ proteins (Figure 6B and Figure S7). A similar in-vivo analysis was attempted using HACC-2A cells. However, there was no evidence of tumor growth even after 3 months from this cell line model. Overall, our data suggests that while MYB-NFIB fusion expressing ACC-01 cells are capable of growing as implanted tumors in vivo, they do not appear to impart growth advantage over the parental or MYB^wt^ cells.

## 4. Discussion

Here we evaluated the identity of MYB breakpoint sites in fusion transcripts from patient-derived xenografts and patient tumors of ACC harboring the t(6;9) translocation, based on which MYB-NFIB expressing system was established for in vitro and in vivo xenograft study model.

Our analysis revealed the presence of multiple MYB junctions and indicated that MYB breakpoint location occurs primarily downstream of exon 8. These observations appear consistent with report by others showing the heterogeneity in MYB breakpoints found in ACC. For example, breakpoints detected in ACC from Drs. Caulin and El-Nagar’s group involved MYB exons 8–11, 13–16 [36,37]. Similarly, others have identified breakpoints at MYB exon 8, 9, 10, 12, and 14 [14,26,38,39]. Comparison between these studies and our data substantiated that MYB breakpoint occurs predominantly downstream of exon 8, and further indicated MYB exon 8 as one of the most frequent breakpoint sites that could form fusion with NFIB. Although the primary patients’ tumor samples referenced in this report were no longer accessible for analysis, one of the patient-derived xenografts utilized in this study (ACCX20M1) represented a case of MYB-NFIB fusion with the MYB exon 8 breakpoint. We utilized it as a template to establish a reliable model of a chimeric MYB-NFIB protein. Additional MYB-NFIB fusions, including variants with MYB breakpoint at exons 9 or 12 were created out of ACCX29 and ACCX11, respectively.

MYB, a transcription regulator essential in normal tissue homeostasis, developmental process, and as oncogenes in leukemias and other solid cancers, consists of an N-terminal DNA-binding domain (DBD), a centrally located transcriptional activation domain (TAD) and a C-terminus negative regulatory domain (NRD) [40,41]. Engagement of DBD to PyAACG/TG consensus sequence and interaction of TAD with co-activator and co-repressor proteins are essential for MYB transcriptional induction or repression; while the NRD harbor sites for post-translational modifications such as, phosphorylation, ubiquitylation, sumoylation and acetylation, that impact MYB’s regulatory activities, including protein stability [42,43,44]. Many of the known MYB-NFIB fusions, including fusions generated in this study, have partial disruption or complete loss of the NRD while retaining a functional DBD and TAD motifs. Indeed, Brayer et al. has confirmed the functional capacity of NRD-lacking fusion proteins by demonstrating that MYB-NFIB, representing MYB breakpoints at exon 8 or 9, highly induced a synthetic 5X-MRE- and KRT16-based gene reporter activities in HEK293T cells [26]. We posited that ectopic expression of MYB-NFIB fusions and analysis in bona fide ACC cell lines may provide further insight into the obscured activities of MYB-NFIB fusions, which otherwise have been limited seemingly due to the lack of reliable ACC models.

The validated ACC-01 and HACC-2A cells utilized in this work, do not either harbor the t(6;9) translocation [21] or express known fusion-specific protein [22], and thus served as a suitable cell line model to study the activities of MYB-NFIB fusion. In a series of biochemical analyses using multiple MYB antibodies, we verified endogenous MYB-NFIB expression in the fusion-positive patient-derived xenografts as well as the exogenously expressed fusions in the two cell lines. Notably, our data demonstrated the delayed proteolytic events of MYB-NFIB fusion relative to a full-length MYB. This observation is reminiscent of the prolonged protein stability displayed by C-terminal-lacking oncogenic MYB variants that are generally found in myeloid leukemias [45]. Defects in post-translational modification of MYB-NFIB may have contributed to its increased protein stability. Concomitantly, it is reasonable to speculate that such structurally modified MYB-NFIB fusion may influence its protein-protein interactions and consequently affect downstream transcriptional activities [17].

Our study showed that ectopic MYB-NFIB expression upregulated several genes associated in interferon-related signaling pathway. This observation is based on the M8:N10 fusion construct and a single PDX (ACCX20M1) harboring identical fusion variant, and we note that whether such observed transcriptional effects depend on specific fusion variant remains to be ascertained. Nevertheless, it is possible that the substitution of MYB C-terminus with the short NFIB fragment modifies MYB-NFIB function leading to downstream transcriptional activities that are distinct from full-length MYB. Previous work involving domain swapping, N-terminal truncation, and sequential deletion of C-terminal region of MYB has shown to yield unique gene expression profiles [46,47,48]. These studies suggest that structural composition flanking the DBD region of MYB may govern the transcriptional regulation of specific target genes. Mechanistically, it remains to be determined whether the lack of MYB domains, encoded by exon 9–16, or the presence of the short NFIB fragment, directly regulates the transcriptional activities of MYB-NFIB fusion observed in ACC cells. Several of the interferon-induced gene functions are not only associated with responses to innate antiviral immunity system but are also implicated in chemoresistance, tumor progression and metastasis. For example, induction of ISG15 or IFIT1 in human squamous cell carcinoma cancer cell line conferred doxorubicin chemo-resistance [49], or overexpression of IFIT1 and IFIT3 in oral squamous cell carcinoma (OSCC) promoted experimental metastasis [50]. The latter study also reported a positive association of heightened IFIT1 and IFIT3 expression to nodal metastasis and perineural invasion in advanced T-stage OSCC patients. Further investigation is clearly warranted to determine the significance of the enriched genes in the context of MYB-NFIB fusion expression in ACC.

We finally evaluated MYB-NFIB expression system and its capacity to promote tumor growth in vivo. When cells were implanted subcutaneously into the flanks of mice, the efficiency of tumor growth formation was comparable between parental, MYB^wt^ and MYB-NFIB cells, suggesting that MYB-NFIB expression by itself does not enhance tumor growth activities. This observation corresponds with our in vitro results showing that MYB-NFIB expression provided no cell growth advantage. Andersson et al. have also reported that MYB variants or MYB-NFIB fusion overexpression is not sufficient for tumor growth and have suggested requiring supplemental signaling mechanisms for tumor formation [51]. Nonetheless, our data established MYB-NFIB expressing ACC cells capable of growing as implanted tumors in vivo. Future work will examine whether MYB-NFIB fusion is involved in promoting tumor metastasis.

## 5. Conclusions

We describe here an inducible MYB-NFIB fusion expression system based on MYB breakpoints identified from primary ACC patient and patient-derived xenograft tumors. Currently, the availability of a reliable ACC cell line model that express the various MYB-NFIB fusion for functional evaluation and other critical pre-clinical studies are limited. We believe that the described expression system may be useful not only in studies to further understand the functional aspects of MYB-NFIB fusion but also crucial in determining the impact of MYB-NFIB fusion in drug response studies.

## Data Availability

All data are available in the text and supplementary.

## Supplementary Materials

The following supporting information can be downloaded at: https://www.mdpi.com/article/10.3390/cancers14092263/s1, Table S1: List of primers used for the cloning and qPCR analysis; Table S2: List of MYB breakpoints identified in MYB-NFIB fusion positive ACC patients using NeoFusion and Arriba tool; Table S3: Differentially expressed genes between MYB vs MYB-NFIB expressing ACC cells; Figure S1: Original Western blots for data shown in Figure 2. Figure S2: Original Western blots for data shown in Figure 3C,D. Figure S3: MYB-NFIB fusion expression in ACCX11 and ACCX20M1; Figure S4: In vitro growth activities of MYB-MNFIB fusion in ACC cells; Figure S5: Original Western blots for data shown in Figure 4A,C,E. Figure S6: Differential protein stability of MYB^wt^ and MYB-NFIB fusion variants. Figure S7: Original Western blots for data shown in Figure 6B.

## References

[1] Seethala R.R., Stenman G. (2017). Update from the 4th Edition of the World Health Organization Classification of Head and Neck Tumours: Tumors of the Salivary Gland. Head Neck Pathol..

[2] Young A., Okuyemi O.T. (2022). Malignant Salivary Gland Tumors.

[3] Spiro R.H. (1997). Distant metastasis in adenoid cystic carcinoma of salivary origin. Am. J. Surg..

[4] Park G., Roh J., Cho K., Jin M., Choi S., Nam S., Kim S. (2017). Incidence and risk factors of late recurrence in patients with salivary gland cancer. Clin. Otolaryngol..

[5] Ohta K., Matsuda S., Okada A., Sasaki M., Imamura Y., Yoshimura H. (2021). Adenoid cystic carcinoma of the sublingual gland developing lung metastasis 20 years after primary treatment. Medicine.

[6] Sahara S., Herzog A.E., Nor J.E. (2021). Systemic therapies for salivary gland adenoid cystic carcinoma. Am. J. Cancer Res..

[7] Geiger J.L., Ismaila N., Beadle B., Caudell J.J., Chau N., Deschler D., Glastonbury C., Kaufman M., Lamarre E., Lau H.Y. (2021). Management of Salivary Gland Malignancy: ASCO Guideline. J. Clin. Oncol..

[8] Mueller S.K., Haderlein M., Lettmaier S., Agaimy A., Haller F., Hecht M., Fietkau R., Iro H., Mantsopoulos K. (2022). Targeted Therapy, Chemotherapy, Immunotherapy and Novel Treatment Options for Different Subtypes of Salivary Gland Cancer. J. Clin. Med..

[9] Zheng J. (2013). Oncogenic chromosomal translocations and human cancer (Review). Oncol. Rep..

[10] Nambiar M., Kari V., Raghavan S.C. (2008). Chromosomal translocations in cancer. Biochim. Biophys. Acta.

[11] Fröhling S., Döhner H. (2008). Chromosomal Abnormalities in Cancer. N. Engl. J. Med..

[12] Meyer M., Watermann C., Dreyer T., Ergün S., Karnati S. (2021). 2021 Update on Diagnostic Markers and Translocation in Salivary Gland Tumors. Int. J. Mol. Sci..

[13] Wysocki P., Izumchenko E., Meir J., Ha P.K., Sidransky D., Brait M. (2016). Adenoid cystic carcinoma: Emerging role of translocations and gene fusions. Oncotarget.

[14] Persson M., Andren Y., Mark J., Horlings H.M., Persson F., Stenman G. (2009). Recurrent fusion of MYB and NFIB transcription factor genes in carcinomas of the breast and head and neck. Proc. Natl. Acad. Sci. USA.

[15] Brill L.B., Kanner W., Fehr A., Andrén Y., Moskaluk C., Löning T., Stenman G., Frierson Jr H.F. (2011). Analysis of MYB expression and MYB-NFIB gene fusions in adenoid cystic carcinoma and other salivary neoplasms. Mod. Pathol..

[16] Drier Y., Cotton M.J., Williamson K.E., Gillespie S., Ryan R., Kluk M.J., Carey C.D., Rodig S.J., Sholl L.M., Afrogheh A.H. (2016). An oncogenic MYB feedback loop drives alternate cell fates in adenoid cystic carcinoma. Nat. Genet..

[17] George O.L., Ness S.A. (2014). Situational Awareness: Regulation of the Myb Transcription Factor in Differentiation, the Cell Cycle and Oncogenesis. Cancers.

[18] Moskaluk C., Baras A.S., Mancuso S., Fan H., Davidson R.J., Dirks D.C., Golden W.L., Jr H.F.F. (2011). Development and characterization of xenograft model systems for adenoid cystic carcinoma. Lab. Investig..

[19] ACCRF PDX Models and Screening Program. https://accrf.org/tools-for-researchers/pdx-models-and-screening-program/.

[20] Humtsoe J.O., Kim H.-S., Leonard B., Ling S., Keam B., Marchionni L., Afsari B., Considine M., Favorov A.V., Fertig E.J. (2021). Newly Identified Members of FGFR1 Splice Variants Engage in Cross-talk with AXL/AKT Axis in Salivary Adenoid Cystic Carcinoma. Cancer Res..

[21] Li J., Perlaky L., Rao P., Weber R.S., El-Naggar A.K. (2014). Development and characterization of salivary adenoid cystic carcinoma cell line. Oral Oncol..

[22] Warner K.A., Oklejas A.E., Pearson A., Zhang Z., Wu W., Divi V., Rodriguez-Ramirez C., Castilho R.M., Polverini P.J., Nör J.E. (2018). UM-HACC-2A: MYB-NFIB fusion-positive human adenoid cystic carcinoma cell line. Oral Oncol..

[23] Rettig E.M., Talbot C.C., Sausen M., Jones S., Bishop J.A., Wood L.D., Tokheim C., Niknafs N., Karchin R., Fertig E. (2016). Whole-Genome Sequencing of Salivary Gland Adenoid Cystic Carcinoma. Cancer Prev. Res..

[24] Fotakis G., Rieder D., Haider M., Trajanoski Z., Finotello F. (2019). NeoFuse: Predicting fusion neoantigens from RNA sequencing data. Bioinformatics.

[25] Uhrig S., Ellermann J., Walther T., Burkhardt P., Fröhlich M., Hutter B., Toprak U.H., Neumann O., Stenzinger A., Scholl C. (2021). Accurate and efficient detection of gene fusions from RNA sequencing data. Genome Res..

[26] Brayer K.J., Frerich C.A., Kang H., Ness S.A. (2015). Recurrent Fusions in MYB and *MYBL1* Define a Common, Transcription Factor–Driven Oncogenic Pathway in Salivary Gland Adenoid Cystic Carcinoma. Cancer Discov..

[27] Dobin A., Davis C.A., Schlesinger F., Drenkow J., Zaleski C., Jha S., Batut P., Chaisson M., Gingeras T.R. (2013). STAR: Ultrafast universal RNA-seq aligner. Bioinformatics.

[28] Love M.I., Huber W., Anders S. (2014). Moderated estimation of fold change and dispersion for RNA-seq data with DESeq2. Genome Biol..

[29] Gentleman R.C., Carey V.J., Bates D.M., Bolstad B., Dettling M., Dudoit S., Ellis B., Gautier L., Ge Y., Gentry J. (2004). Bioconductor: Open software development for computational biology and bioinformatics. Genome Biol..

[30] Zhu A., Ibrahim J.G., Love M. (2018). Heavy-tailed prior distributions for sequence count data: Removing the noise and preserving large differences. Bioinformatics.

[31] Croft D., Mundo A.F., Haw R., Orlic-Milacic M., Weiser J., Wu G., Caudy M., Garapati P.V., Gillespie M., Kamdar M.R. (2013). The Reactome pathway knowledgebase. Nucleic Acids Res..

[32] Yu G., He Q.-Y. (2015). ReactomePA: An R/Bioconductor package for reactome pathway analysis and visualization. Mol. BioSyst..

[33] Panaccione A., Zhang Y., Ryan M., Moskaluk C.A., Anderson K.S., Yarbrough W.G., Ivanov S.V. (2017). MYB fusions and CD markers as tools for authentication and purification of cancer stem cells from salivary adenoid cystic carcinoma. Stem Cell Res..

[34] Diamond M.S., Farzan M. (2013). The broad-spectrum antiviral functions of IFIT and IFITM proteins. Nat. Rev. Immunol..

[35] Schneider W.M., Chevillotte M.D., Rice C.M. (2014). Interferon-Stimulated Genes: A Complex Web of Host Defenses. Annu. Rev. Immunol..

[36] Mitani Y., Li J., Rao P.H., Zhao Y.-J., Bell D., Lippman S.M., Weber R.S., Caulin C., El-Naggar A.K. (2010). Comprehensive Analysis of the MYB-NFIB Gene Fusion in Salivary Adenoid Cystic Carcinoma: Incidence, Variability, and Clinicopathologic Significance. Clin. Cancer Res..

[37] Mitani Y., Liu B., Rao P.H., Borra V.J., Zafereo M., Weber R.S., Kies M., Lozano G., Futreal P.A., Caulin C. (2015). Novel *MYBL1* Gene Rearrangements with Recurrent *MYBL1–NFIB* Fusions in Salivary Adenoid Cystic Carcinomas Lacking t(6;9) Translocations. Clin. Cancer Res..

[38] Togashi Y., Dobashi A., Sakata S., Sato Y., Baba S., Seto A., Mitani H., Kawabata K., Takeuchi K. (2018). MYB and MYBL1 in adenoid cystic carcinoma: Diversity in the mode of genomic rearrangement and transcripts. Mod. Pathol..

[39] Yang W., Lee K.-W., Srivastava R.M., Kuo F., Krishna C., Chowell D., Makarov V., Hoen D., Dalin M.G., Wexler L. (2019). Immunogenic neoantigens derived from gene fusions stimulate T cell responses. Nat. Med..

[40] Ramsay R.G., Gonda T.J. (2008). MYB function in normal and cancer cells. Nat. Cancer.

[41] Ness S. (1999). Myb binding proteins: Regulators and cohorts in transformation. Oncogene.

[42] Wang X., Angelis N., Thein S.L. (2018). MYB—A regulatory factor in hematopoiesis. Gene.

[43] Kitagawa K., Hiramatsu Y., Uchida C., Isobe T., Hattori T., Oda T., Shibata K., Nakamura S., Kikuchi A. (2009). Fbw7 promotes ubiquitin-dependent degradation of c-Myb: Involvement of GSK3-mediated phosphorylation of Thr-572 in mouse c-Myb. Oncogene.

[44] Bies J., Feiková S., Markus J., Wolff L. (2001). Phosphorylation-Dependent Conformation and Proteolytic Stability of c-Myb. Blood Cells Mol. Dis..

[45] Bies J., Wolff L. (1997). Oncogenic activation of c-Myb by carboxyl-terminal truncation leads to decreased proteolysis by the ubiquitin-26S proteasome pathway. Oncogene.

[46] Lei W., Rushton J.J., Davis L.M., Liu F., Ness S. (2004). Positive and Negative Determinants of Target Gene Specificity in Myb Transcription Factors. J. Biol. Chem..

[47] Liu F., Lei W., O’Rourke J.P., Ness S. (2005). Oncogenic mutations cause dramatic, qualitative changes in the transcriptional activity of c-Myb. Oncogene.

[48] Frerich C.A., Sedam H.N., Kang H., Mitani Y., El-Naggar A.K., Ness S.A. (2019). N-Terminal Truncated Myb with New Transcriptional Activity Produced Through Use of an Alternative MYB Promoter in Salivary Gland Adenoid Cystic Carcinoma. Cancers.

[49] Weichselbaum R.R., Ishwaran H., Yoon T., Nuyten D.S.A., Baker S.W., Khodarev N., Su A.W., Shaikh A.Y., Roach P., Kreike B. (2008). An interferon-related gene signature for DNA damage resistance is a predictive marker for chemotherapy and radiation for breast cancer. Proc. Natl. Acad. Sci. USA.

[50] Pidugu V.K., Wu M.-M., Yen A.-H., Pidugu H.B., Chang K.-W., Liu C.-J., Lee T.-C. (2019). IFIT1 and IFIT3 promote oral squamous cell carcinoma metastasis and contribute to the anti-tumor effect of gefitinib via enhancing p-EGFR recycling. Oncogene.

[51] Andersson M.K., Mangiapane G., Nevado P.T., Tsakaneli A., Carlsson T., Corda G., Nieddu V., Abrahamian C., Chayka O., Rai L. (2020). ATR is a MYB regulated gene and potential therapeutic target in adenoid cystic carcinoma. Oncogenesis.

