# Supplementary material for: Development and Characterization of MYB-NFIB Fusion Expression in Adenoid Cystic Carcinoma
Source: Cancers (Basel). 2022 Apr 30;14(9):2263. doi: 10.3390/cancers14092263 (PMC9103462; doi:10.3390/cancers14092263)
Supplement: Supplementary file 1 [file cancers-14-02263-s001.zip › cancers-1692334-supplementary.pdf]

Supplemental Table S1: List of primers used for the cloning and qPCR analysis.

		Primers used for MYB-NFIB fusion detection and cloning			
**Primer ID**	**Direction**	**Sequence (5'- 3')**	***Gene***	**Target**	**Source**
Mex6F	forward	5’-CCAGCAGTGGCCACAAGCTT-3’	*MYB*	exon 6	Sigma-Aldrich
Mex9R	reverse	5’- ATCATGCACCTTGCTGGCGA-3’	*MYB*	exon 9	Sigma-Aldrich
Nex11R	reverse	5’- TGGCCGGTAAGATGGGTGTC-3’	*NFIB*	exon 11	Sigma-Aldrich
MYB-orf-F	forward	5’- ATCAACCTGTTTCCTCCTCC-3’	*MYB*	exon 1	IDT, Inc.
NFIB-orf-R	reverse	5’- GCCTTTGTGTTGTTTTGTCCA-3’	*NFIB*	exon 12	IDT, Inc.
attMYB-F	forward	5’- ggggacaagtttgtacaaaaaagcaggcttCACCATGGCCCGAAGACCCCGA -3’	*MYB*	exon 1	Sigma-Aldrich
attMYB-R	reverse	5’-ggggaccactttgtacaagaaagctgggttCATGACCAGCGTCCGGGC -3’	*MYB*	exon 16	Sigma-Aldrich
attNFIB-R	reverse	5’-ggggaccactttgtacaagaaagctgggttGTTGCTTGTTTCTGCTTG -3’	*NFIB*	exon 12	Sigma-Aldrich
attNFIB-R^{ex11}	reverse	5’-ggggaccactttgtacaagaaagctgggttGCCCAGGTACCAGGACTC -3’	*NFIB*	exon 11	Sigma-Aldrich
		**Primers used for RT qPCR**			
**Primer ID**		**Sequence (Forward: Reverse)**	**Gene**	**source**	
OAS1		5'-TGTCCAAGGTGGTAAAGGGTG-3': 5'-CCGGCGATTTAACTGATCCTG-3'	*OAS1*	Sigma-Aldrich	
OAS2		5'-CTCAGAAGCTGGGTTGGTTTAT-3': 5'-ACCATCTCGTCGATCAGTGTC-3'	*OAS2*	Sigma-Aldrich	
OAS3		5'-GAAGGAGTTCGTAGAGAAGGCG-3': 5'-CCCTTGACAGTTTTCAGCACC-3'	*OAS3*	Sigma-Aldrich	
OASL		5'-CTGATGCAGGAACTGTATAGCAC-3': 5'-CACAGCGTCTAGCACCTCTT-3'	*OASL*	Sigma-Aldrich	
GBP4		5'-ATGGGTGAGAGAACTCTTCACG-3': 5'-TGCGGTATAGCCCTACAATGG-3'	*GBP4*	Sigma-Aldrich	
GBP5		5'-CCATGTGCCTCATCGAGAACT-3': 5'-ACAGGTTGCGTAATGGCAGAC-3'	*GBP5*	Sigma-Aldrich	
IFI27		5'-TGCTCTCACCTCATCAGCAGT-3': 5'-CACAACTCCTCCAATCACAACT-3'	*IFI27*	Sigma-Aldrich	
BST2		5'-CACACTGTGATGGCCCTAATG-3': 5'-GTCCGCGATTCTCACGCTT-3'	*BST2*	Sigma-Aldrich	
IFI6		5'-GGTCTGCGATCCTGAATGGG-3': 5'-TCACTATCGAGATACTTGTGGGT-3'	*IFI6*	Sigma-Aldrich	
XAF1		5'-GCTCCACGAGTCCTACTGTG-3': 5'-GTTCACTGCGACAGACATCTC-3'	*XAF1*	Sigma-Aldrich	
IRF7		5'-GCTGGACGTGACCATCATGTA-3': 5'-GGGCCGTATAGGAACGTGC-3'	*IRF7*	Sigma-Aldrich	
HERC5		5'-GGTGAGCTTTTTGCCTGGG-3': 5'-TTCTCCGGCAGAAATCTGAGC-3'	*HERC5*	Sigma-Aldrich	
DDX58		5'-CTGGACCCTACCTACATCCTG-3': 5'-GGCATCCAAAAAGCCACGG-3'	*DDX58*	Sigma-Aldrich	
ISG15		5'-CGCAGATCACCCAGAAGATCG-3': 5'-TTCGTCGCATTTGTCCACCA-3'	*ISG15*	Sigma-Aldrich	
MX1		5'-GTTTCCGAAGTGGACATCGCA-3': 5'-CTGCACAGGTTGTTCTCAGC-3'	*MX1*	Sigma-Aldrich	
MX2		5'-CAGAGGCAGCGGAATCGTAA-3': 5'-TGAAGCTCTAGCTCGGTGTTC-3'	*MX2*	Sigma-Aldrich	
IFIT1		5'-TTGATGACGATGAAATGCCTGA-3': 5'-CAGGTCACCAGACTCCTCAC-3'	*IFIT1*	Sigma-Aldrich	
IFIH1		5'-TCGAATGGGTATTCCACAGACG-3': 5'-GTGGCGACTGTCCTCTGAA-3'	*IFIH1*	Sigma-Aldrich	
USP18		5'-CCTGAGGCAAATCTGTCAGTC-3': 5'-CGAACACCTGAATCAAGGAGTTA-3'	*USP18*	Sigma-Aldrich	
TBP		5'-CCCATGACTCCCATGACC-3': 5'-TTTACAACCAAGATTCACTGTGG-3'	*TBP*	Sigma-Aldrich	

Supplementary Table S2: Identification of MYB breakpoints in MYB-NFIB fusion positive ACC patients using NeoFusion and Arriba tool.

Patient #	patient sample_ic	gene1	gene2	*MYB* breakpoint	*MYB* exon C'-position	*NFIB* breakpoint	*NFIB* exon N'-position	site1	site2	type	direction1	direction2	split_reads1	split_reads2	discordant_mates	coverage1	coverage2	confidence
1	HN335PT_30	MYB	NFIB	6:135524462	Exon 15	9:14084728	downstream of stop codon	splice-site	3'UTR	translocation	downstream	downstream	145	79	30	291	544	high
2	HN348PT_20	MYB	NFIB	6:135515598	Exon 8	9:14088325	Exon 11	splice-site	splice-site	translocation	downstream	downstream	57	72	29	642	563	high
	HN348PT_20	MYB	NFIB	6:135515589	Exon 8s	9:14088325	Exon 11	splice-site	splice-site	translocation	downstream	downstream	19	29	22	642	563	high
	HN348PT_20	MYB	NFIB	6:135516219	Intron 8	9:14088325	Exon 11	splice-site	splice-site	translocation	downstream	downstream	1	4	31	125	563	high
	HN348PT_20	MYB	NFIB	6:135515056	Exon 7	9:14088325	Exon 11	splice-site	splice-site	translocation	downstream	downstream	0	1	3	866	563	medium
3	HN341PT_17	MYB	NFIB	6:135524462	Exon 15	9:14102509	Exon 10	splice-site	splice-site	translocation	downstream	downstream	124	56	24	302	385	high
	HN341PT_17	MYB	NFIB	6:135524462	Exon 15	9:14088325	Exon 11	splice-site	splice-site	translocation	downstream	downstream	2	3	11	302	799	high
4	HN312PT_22	MYB	NFIB	6:135515598	Exon 8	9:14102509	Exon 10	splice-site	splice-site	translocation	downstream	downstream	89	61	30	694	377	high
	HN312PT_22	MYB	NFIB	6:135515589	Exon 8s	9:14102509	Exon 10	splice-site	splice-site	translocation	downstream	downstream	44	16	23	694	377	high
	HN312PT_22	MYB	NFIB	6:135515598	Exon 8	9:14088325	Exon 11	splice-site	splice-site	translocation	downstream	downstream	1	5	5	694	723	high
	HN312PT_22	MYB	NFIB	6:135515589	Exon 8s	9:14088325	Exon 11	splice-site	splice-site	translocation	downstream	downstream	1	1	4	694	723	high
5	HN345PT_19	MYB	NFIB	6:135524462	Exon 15	9:14102509	Exon 10	splice-site	splice-site	translocation	downstream	downstream	27	10	13	114	233	high
	HN345PT_19	MYB	NFIB	6:135524462	Exon 15	9:14113080	Exon 9	splice-site	splice-site	translocation	downstream	downstream	8	11	24	114	190	high
	HN345PT_19	MYB	NFIB	6:135524462	Exon 15	9:14088325	Exon 11	splice-site	splice-site	translocation	downstream	downstream	8	15	7	114	417	high
6	HN334PT_29	MYB	NFIB	6:135539114	Exon 16	9:14088325	Exon 11	CDS	splice-site	translocation	downstream	downstream	61	112	33	349	552	high
7	HN338PT_16	MYB	NFIB	6:135520188	Ex11	9:14088325	Exon 11	splice-site	splice-site	translocation	downstream	downstream	96	104	47	311	681	high
8	HN325PT_26	MYB	NFIB	6:135515598	Exon 8	9:14088325	Exon 11	splice-site	splice-site	translocation	downstream	downstream	47	70	36	740	569	high
	HN325PT_26	MYB	NFIB	6:135515589	Exon 8s	9:14088325	Exon 11	splice-site	splice-site	translocation	downstream	downstream	14	25	32	740	569	high
	HN325PT_26	MYB	NFIB	6:135516219	Intron 8	9:14088325	Exon 11	splice-site	splice-site	translocation	downstream	downstream	2	2	37	122	569	high
	HN325PT_26	MYB	NFIB	6:135515056	Exon 7	9:14088325	Exon 11	splice-site	splice-site	translocation	downstream	downstream	0	2	4	1069	569	medium
9	HN317PT_23	MYB	NFIB	6:135524462	Exon 15	9:14102509	Exon 10	splice-site	splice-site	translocation	downstream	downstream	48	24	18	199	220	high
	HN317PT_23	MYB	NFIB	6:135524462	Exon 15	9:14088325	Exon 11	splice-site	splice-site	translocation	downstream	downstream	15	24	6	199	514	high
	HN317PT_23	MYB	NFIB	6:135524462	Exon 15	9:14113080	Exon 9	splice-site	splice-site	translocation	downstream	downstream	6	11	23	199	189	high
10	HN332PT_27	MYB	NFIB	6:135515598	Exon 8	9:14088325	Exon 11	splice-site	splice-site	translocation	downstream	downstream	16	18	15	781	360	high
	HN332PT_27	MYB	NFIB	6:135515589	Exon 8s	9:14088325	Exon 11	splice-site	splice-site	translocation	downstream	downstream	1	5	13	781	360	high
	HN332PT_27	MYB	NFIB	6:135516219	Intron 8	9:14088325	Exon 11	splice-site	splice-site	translocation	downstream	downstream	0	1	15	69	360	high

Exon 8s: lacks 9 nucleotides at the exon 8 splice site.

Supplementary Table S3: Differentially expressed genes between MYB vs MYB-NFIB. After adjusting for multiple testing (FDR <0.05), differentially expressed genes were called by applying a fold-change cutoff of two.

		symbol	baseMean	log2FoldChange	lfcSE	pvalue	padj
1	ENSG00000004809	SLC22A16	5.017817387	-6.712606466	1.15053973	5.40E-09	2.47E-07
2	ENSG00000254146	HMGB1P46	4.863668187	-6.66517831	1.16097046	9.41E-09	4.07E-07
3	ENSG00000118308	IRAG2	22.73876629	-4.076651897	0.42303635	5.60E-22	1.27E-19
4	ENSG00000165983	PTER	12.8070835	-4.064277435	0.53995906	5.19E-14	4.62E-12
5	ENSG00000144057	ST6GAL2	13.73707483	-4.047540347	0.55484542	2.99E-13	2.43E-11
6	ENSG00000169122	FAM110B	5.766748922	-4.035289908	0.81896469	8.34E-07	2.65E-05
7	ENSG00000169862	CTNND2	14.42126349	-3.550789491	1.20858192	0.00330358	0.03301126
8	ENSG00000099399	MAGEB2	152.9724349	-3.449339145	0.14478291	1.87E-125	6.04E-122
9	ENSG00000022556	NLRP2	27.74923978	-3.407480039	0.35300181	4.78E-22	1.12E-19
10	ENSG00000060303	RPS17P5	14.78560872	-3.238143834	0.46320342	2.73E-12	1.96E-10
11	ENSG00000227234	SPANXB1	11.41255648	-3.214175135	0.50023211	1.32E-10	7.68E-09
12	ENSG00000111837	MAK	8.335021505	-3.167597604	0.60923771	2.00E-07	7.26E-06
13	ENSG00000089177	KIF16B	58.85440306	-3.094999725	0.22103592	1.51E-44	1.43E-41
14	ENSG00000196406	SPANXD	78.08712272	-3.002821564	0.18674148	3.52E-58	6.30E-55
15	ENSG00000081237	PTPRC	7.066407771	-2.950318442	0.67103785	1.10E-05	0.00027219
16	ENSG00000132972	RNF17	11.49575718	-2.937719865	0.49284648	2.51E-09	1.19E-07
17	ENSG00000230316	FEZF1-AS1	9.250077703	-2.909400519	0.54187913	7.91E-08	3.03E-06
18	ENSG00000197077	KIAA1671	61.17331796	-2.884195777	0.21741994	3.67E-40	2.36E-37
19	ENSG00000149557	FEZ1	24.5978393	-2.812782227	0.32471456	4.62E-18	6.77E-16
20	ENSG00000183166	CALN1	36.96930154	-2.791473379	0.28480374	1.11E-22	2.75E-20
21	ENSG00000179163	FUCA1	30.90234101	-2.738054503	0.28410117	5.55E-22	1.27E-19
22	ENSG00000144908	ALDH1L1	67.04846338	-2.668214132	0.20636625	3.07E-38	1.90E-35
23	ENSG00000172543	CTSW	6.95652474	-2.56072554	0.61332794	2.98E-05	0.00064955
24	ENSG00000144229	THSD7B	17.09918251	-2.523489978	0.41736291	1.48E-09	7.30E-08
25	ENSG00000101850	GPR143	10.12128629	-2.513358621	0.51335535	9.78E-07	3.03E-05
26	ENSG00000080166	DCT	7.217116048	-2.428185854	0.62369639	9.89E-05	0.00184116
27	ENSG00000248964	LOC101929470	9.451779055	-2.365495917	0.50620225	2.97E-06	8.36E-05
28	ENSG00000121316	PLBD1	59.82451017	-2.349668874	0.19581143	3.57E-33	1.74E-30
29	ENSG00000077092	RARB	8.516121821	-2.339914921	0.5406316	1.50E-05	0.00035858
30	ENSG00000234722	LINC01287	64.55862736	-2.327747844	0.19051774	2.49E-34	1.38E-31
31	ENSG00000130477	UNC13A	54.80888495	-2.27176421	0.21938487	3.97E-25	1.23E-22
32	ENSG00000023892	DEF6	31.30976227	-2.268725182	0.39233578	7.36E-09	3.27E-07
33	ENSG00000169164	XAGE-4	6.355493143	-2.265593412	0.5954633	0.00014194	0.00251965
34	ENSG00000225756	DBH-AS1	9.228373164	-2.219591155	0.51228023	1.47E-05	0.00035265
35	ENSG00000093072	ADA2	11.92451253	-2.216028208	0.52608043	2.53E-05	0.00056344
36	ENSG00000104313	EYA1	47.2430923	-2.213296556	0.21926135	5.85E-24	1.63E-21
37	ENSG00000060718	COL11A1	407.0888502	-2.208704546	0.08745804	1.01E-140	5.44E-137
38	ENSG00000152402	GUCY1A2	41.17037279	-2.18387572	0.25324373	6.49E-18	9.09E-16
39	ENSG00000081052	COL4A4	92.83170501	-2.13575188	0.22185214	6.15E-22	1.38E-19
40	ENSG00000123360	PDE1B	15.27726721	-2.123623731	0.41989378	4.25E-07	1.45E-05
41	ENSG00000169064	ZBBX	8.614764149	-2.123408601	0.52858251	5.89E-05	0.00118669
42	ENSG00000072858	SIDT1	31.29130004	-2.11616292	0.27770492	2.53E-14	2.32E-12
43	ENSG00000169174	PCSK9	57.37871818	-2.099271488	0.19802787	2.95E-26	9.80E-24
44	ENSG00000116703	PDC	47.99021499	-2.085449204	0.23682459	1.30E-18	2.05E-16
45	ENSG00000189299	FOXR2	20.12727361	-2.063530297	0.34130891	1.49E-09	7.30E-08
46	ENSG00000249948	GBA3	96.41060469	-2.054706008	0.15065086	2.35E-42	2.00E-39
47	ENSG00000177098	SCN4B	13.4327883	-2.024052813	0.40037968	4.30E-07	1.46E-05
48	ENSG00000133056	PIK3C2B	17.46974091	-1.96876092	0.45484091	1.50E-05	0.00035855
49	ENSG00000183844	FAM3B	37.5948877	-1.959930232	0.2420487	5.62E-16	6.51E-14
50	ENSG00000238269	PAGE2B	28.93086627	-1.955598798	0.29260933	2.34E-11	1.49E-09
51	ENSG00000213315	RPS18P2	7.528293615	-1.950426003	0.60446426	0.00125226	0.01534897
52	ENSG00000187242	KRT12	9.49903222	-1.948047221	0.50490053	0.00011419	0.00209622
53	ENSG00000128564	VGF	27.59953053	-1.933197265	0.28855719	2.09E-11	1.34E-09
54	ENSG00000133454	MYO18B	25.77480135	-1.92447712	0.32430706	2.95E-09	1.39E-07
55	ENSG00000183434	TFDP3	8.881684009	-1.870835135	0.48642148	0.00012	0.00218308
56	ENSG00000101670	LIPG	10.67437507	-1.864983848	0.44194296	2.44E-05	0.00054927
57	ENSG00000156466	GDF6	16.3362028	-1.864947327	0.36130625	2.45E-07	8.73E-06
58	ENSG00000128606	LRRC17	56.63567007	-1.860431478	0.20078225	1.93E-20	3.90E-18
59	ENSG00000164199	ADGRV1	12.73515734	-1.846881002	0.43142424	1.86E-05	0.0004317
60	ENSG00000171951	SCG2	195.5154993	-1.805886124	0.11752386	2.76E-53	3.71E-50
61	ENSG00000119121	TRPM6	10.69068509	-1.800884144	0.47725451	0.00016102	0.00279671
62	ENSG00000143603	KCNN3	19.0432177	-1.788361935	0.32095729	2.52E-08	1.01E-06
63	ENSG00000139304	PTPRQ	14.12914974	-1.780245983	0.39975259	8.45E-06	0.00021491
64	ENSG00000134538	SLCO1B1	10.07389153	-1.7455175	0.59385909	0.00328977	0.03289361
65	ENSG00000221866	PLXNA4	9.789119665	-1.737238016	0.4657352	0.00019141	0.00322032
66	ENSG00000155265	GOLGA7B	7.442739371	-1.73311621	0.52010082	0.00086141	0.01126047
67	ENSG00000233997	LINC01425	135.0974355	-1.691259194	0.12411898	2.80E-42	2.26E-39
68	ENSG00000118432	CNR1	10.91977885	-1.658270616	0.43467797	0.00013622	0.00243137
69	ENSG00000198185	ZNF334	13.93122816	-1.628236941	0.3860092	2.46E-05	0.00055299
70	ENSG00000068985	PAGE1	251.1305199	-1.625413389	0.09637528	8.08E-64	1.86E-60
71	ENSG00000155846	PPARGC1B	21.6754671	-1.60376206	0.47678014	0.00076895	0.01030257
72	ENSG00000164796	CSMD3	78.19729156	-1.601411467	0.16849031	2.01E-21	4.44E-19
73	ENSG00000066032	CTNNA2	15.20803159	-1.599729415	0.37164972	1.67E-05	0.00039398
74	ENSG00000234068	PAGE2	31.60474167	-1.599222673	0.26711923	2.14E-09	1.02E-07
75	ENSG00000233098	LOC339260	7.853236448	-1.545038723	0.54329677	0.00445761	0.04182057

Supplementary Table S3: Contd:

76	ENSG00000026751	SLAMF7	43.01543841	-1.542427918	0.21004104	2.08E-13	1.76E-11
77	ENSG00000172346	CSDC2	18.95663438	-1.541886826	0.36000167	1.84E-05	0.00042824
78	ENSG00000255099	LOC644335	9.203285859	-1.539530811	0.50477929	0.00228911	0.02443434
79	ENSG00000079308	TNS1	380.0211752	-1.520379115	0.0828204	2.87E-75	7.72E-72
80	ENSG00000171631	P2RY6	11.38113951	-1.516436569	0.48309408	0.0016952	0.01943335
81	ENSG00000204019	CT83	94.94254632	-1.507894876	0.24982861	1.58E-09	7.73E-08
82	ENSG00000189369	GSPT2	42.09479629	-1.495589889	0.22702936	4.47E-11	2.76E-09
83	ENSG00000204832	ST8SIA6-AS1	26.96191111	-1.493907488	0.39611075	0.00016231	0.00280998
84	ENSG00000140465	CYP1A1	88.50661331	-1.486944898	0.16501099	2.04E-19	3.69E-17
85	ENSG00000238265	LINC00317	69.42614877	-1.486534967	0.17005014	2.30E-18	3.52E-16
86	ENSG00000152990	ADGRA3	309.7435368	-1.467863454	0.09121311	2.87E-58	5.79E-55
87	ENSG00000127533	F2RL3	16.10551186	-1.453327934	0.38601553	0.00016659	0.00287474
88	ENSG00000132122	SPATA6	13.06893549	-1.443544511	0.38269697	0.00016193	0.00280937
89	ENSG00000100234	TIMP3	12.92733224	-1.44033295	0.39664221	0.00028198	0.0044646
90	ENSG00000133048	CHI3L1	274.1454059	-1.43989896	0.10662413	1.47E-41	9.89E-39
91	ENSG00000187398	LUZP2	113.6308234	-1.431143092	0.14099803	3.31E-24	9.36E-22
92	ENSG00000163947	ARHGEF3	9.174006329	-1.421108096	0.46279526	0.00213553	0.02332015
93	ENSG00000251191	LINC00589	11.69854983	-1.418540414	0.47014383	0.00255081	0.0266974
94	ENSG00000109771	LRP2BP	20.3566237	-1.40631345	0.34112318	3.75E-05	0.0007966
95	ENSG00000198033	TUBA3C	10.24937129	-1.389822738	0.44055695	0.00160666	0.01858711
96	ENSG00000253301	LINC01606	15.9560152	-1.3837344	0.36420338	0.00014508	0.00256965
97	ENSG00000182492	BGN	53.76409128	-1.332510935	0.23633904	1.72E-08	7.07E-07
98	ENSG00000117215	PLA2G2D	15.23323377	-1.329408702	0.37911248	0.00045381	0.00670445
99	ENSG00000197046	SIGLEC15	117.6260527	-1.326362023	0.1550245	1.17E-17	1.57E-15
100	ENSG00000166689	PLEKHA7	10.63388984	-1.326186639	0.44268967	0.00273773	0.02814201
101	ENSG00000213401	MAGEA12	25.54303113	-1.325375059	0.2759163	1.56E-06	4.70E-05
102	ENSG00000165509	MAGEC3	37.20520932	-1.310977725	0.22748228	8.26E-09	3.64E-07
103	ENSG00000224982	TMEM233	34.30160792	-1.293741527	0.25346195	3.32E-07	1.16E-05
104	ENSG00000066056	TIE1	91.50901547	-1.27570934	0.14759593	5.46E-18	7.79E-16
105	ENSG00000135439	AGAP2	206.3863173	-1.257733413	0.12535286	1.09E-23	2.92E-21
106	ENSG00000233521	LINC01638	11.75048744	-1.245525307	0.41540101	0.00271429	0.02798246
107	ENSG00000143772	ITPKB	64.79911489	-1.240112678	0.18500055	2.04E-11	1.31E-09
108	ENSG00000103942	HOMER2	14.62789849	-1.221624208	0.39400043	0.00193151	0.02155965
109	ENSG00000147041	SYTL5	14.36698093	-1.208653138	0.37982157	0.00146181	0.01733733
110	ENSG00000003137	CYP26B1	17.95076402	-1.203741322	0.35540874	0.00070682	0.00962049
111	ENSG00000213931	HBE1	125.1394794	-1.198907113	0.13367045	2.99E-19	5.35E-17
112	ENSG00000054690	PLEKHH1	19.2518253	-1.187364506	0.32651052	0.00027634	0.00439252
113	ENSG00000155622	XAGE2	11.19209053	-1.172340537	0.40658315	0.00393412	0.03794722
114	ENSG00000167850	CD300C	14.43089041	-1.158927321	0.37421078	0.00195496	0.02171609
115	ENSG00000147443	DOK2	20.16967727	-1.155462081	0.33329842	0.00052679	0.00757783
116	ENSG00000188511	MIR3667HG	14.95782277	-1.144976711	0.39386911	0.00364916	0.03579859
117	ENSG00000145934	TENM2	183.8345987	-1.124320143	0.11550921	2.17E-22	5.14E-20
118	ENSG00000144712	CAND2	98.27440087	-1.119913408	0.17695984	2.47E-10	1.40E-08
119	ENSG00000114487	MORC1	42.96478866	-1.118634615	0.23072809	1.25E-06	3.80E-05
120	ENSG00000151117	TMEM86A	46.2851824	-1.118440618	0.20178504	2.98E-08	1.19E-06
121	ENSG00000151224	MAT1A	30.68211562	-1.109133974	0.26882601	3.69E-05	0.00078648
122	ENSG00000160588	MPZL3	14.00337555	-1.104807357	0.366511	0.00257496	0.02686291
123	ENSG00000169551	CT55	100.627085	-1.092924819	0.19600903	2.46E-08	9.97E-07
124	ENSG00000221852	KRTAP1-5	41.77207734	-1.07154598	0.25844261	3.38E-05	0.00072658
125	ENSG00000107165	TYRP1	124.1724628	-1.06456479	0.14469828	1.88E-13	1.59E-11
126	ENSG00000114013	CD86	15.4074978	-1.032983813	0.37151488	0.00542813	0.04876841
127	ENSG00000171234	UGT2B7	483.3734755	-1.032630111	0.09474414	1.16E-27	4.46E-25
128	ENSG00000077063	CTTNBP2	83.42555747	-1.011212383	0.17426062	6.52E-09	2.92E-07
129	ENSG00000113645	WWC1	57.62727544	-1.009445773	0.22034673	4.62E-06	0.00012318
130	ENSG00000154451	GBP5	1245.782732	0.998716977	0.12430968	9.43E-16	1.03E-13
131	ENSG00000205413	SAMD9	9235.254268	1.000286349	0.17625075	1.38E-08	5.85E-07
132	ENSG00000178685	PARP10	1581.748995	1.0027906	0.14137911	1.31E-12	9.75E-11
133	ENSG00000130775	THEMIS2	379.0646761	1.006373825	0.12202503	1.62E-16	1.98E-14
134	ENSG00000165495	PKNOX2	37.45092	1.008531845	0.23844764	2.34E-05	0.00053077
135	ENSG00000162654	GBP4	578.1635417	1.016537799	0.20316575	5.63E-07	1.86E-05
136	ENSG00000163568	AIM2	44.409172	1.021743866	0.22305631	4.64E-06	0.00012318
137	ENSG00000130589	HELZ2	5708.983003	1.025870851	0.14674091	2.73E-12	1.96E-10
138	ENSG00000115271	GCA	98.55055951	1.026101022	0.16382396	3.77E-10	2.04E-08
139	ENSG00000188313	PLSCR1	5463.677615	1.029134479	0.12396174	1.02E-16	1.28E-14
140	ENSG00000151834	GABRA2	41.00147902	1.041010241	0.2366361	1.09E-05	0.00026942
141	ENSG00000168843	FSTL5	46.32651825	1.056118033	0.25004127	2.40E-05	0.00054157
142	ENSG00000109756	RAPGEF2	4378.935804	1.056468325	0.04396664	1.39E-127	5.60E-124
143	ENSG00000151952	TMEM132D	46.0771148	1.075677501	0.25022956	1.72E-05	0.00040296
144	ENSG00000138642	HERC6	3415.590294	1.076847044	0.13922849	1.04E-14	9.74E-13
145	ENSG00000102524	TNFSF13B	252.6580078	1.082078789	0.13374772	5.94E-16	6.80E-14
146	ENSG00000111199	TRPV4	34.19698977	1.100647222	0.39749504	0.00562358	0.04996742
147	ENSG00000185352	HS6ST3	33.82144868	1.103800728	0.28746028	0.00012312	0.00222714
148	ENSG00000108381	ASPA	28.1849589	1.107026613	0.27489162	5.65E-05	0.00114911
149	ENSG00000115267	IFIH1	1373.161833	1.128167221	0.16983078	3.08E-11	1.92E-09
150	ENSG00000168062	BATF2	237.2286314	1.128243303	0.14533367	8.29E-15	7.90E-13

Supplementary Table S3: Contd:

151	ENSG00000107201	DDX58	4227.220347	1.14288787	0.18200661	3.40E-10	1.86E-08
152	ENSG00000172183	ISG20	95.38628637	1.15762553	0.22700721	3.41E-07	1.19E-05
153	ENSG00000137965	IFI44	2622.842329	1.171583766	0.12366219	2.69E-21	5.86E-19
154	ENSG00000084453	SLCO1A2	32.94004675	1.182226991	0.28479548	3.31E-05	0.00071285
155	ENSG00000153993	SEMA3D	19.92442062	1.195720886	0.36724325	0.00113019	0.01410784
156	ENSG00000133106	EPSTI1	1157.459181	1.213695512	0.15097132	9.04E-16	9.98E-14
157	ENSG00000196220	SRGAP3	550.2922203	1.223985336	0.09012125	5.15E-42	3.78E-39
158	ENSG00000126561	STAT5A	17.2628258	1.254560314	0.37544374	0.00083315	0.01100714
159	ENSG00000133561	GIMAP6	17.72589053	1.25921771	0.34696936	0.00028431	0.0044971
160	ENSG00000078487	ZCWPW1	171.7036457	1.261466608	0.12137935	2.67E-25	8.45E-23
161	ENSG00000174448	STARD6	17.85145483	1.279636441	0.39303694	0.00113087	0.01410784
162	ENSG00000183486	MX2	2830.951602	1.295094577	0.22880832	1.51E-08	6.30E-07
163	ENSG00000229292	RFPL4AL1	10.23949886	1.313964628	0.46754397	0.00494869	0.04539722
164	ENSG00000121858	TNFSF10	254.3969101	1.31989155	0.35285802	0.0001836	0.00311831
165	ENSG00000138646	HERC5	154.764679	1.324348817	0.18684687	1.36E-12	1.01E-10
166	ENSG00000120262	CCDC170	41.646166	1.334629297	0.27156856	8.90E-07	2.81E-05
167	ENSG00000187608	ISG15	3329.680937	1.34873945	0.14664926	3.68E-20	7.15E-18
168	ENSG00000146374	RSPO3	56.84118923	1.350708381	0.23654499	1.13E-08	4.84E-07
169	ENSG00000124875	CXCL6	26.2120512	1.374966653	0.2972654	3.74E-06	0.00010249
170	ENSG00000115738	ID2	114.5944343	1.380990839	0.15735271	1.69E-18	2.64E-16
171	ENSG00000111335	OAS2	5246.250826	1.416308946	0.23380966	1.38E-09	6.90E-08
172	ENSG00000005108	THSD7A	126.1391828	1.418562592	0.16197276	1.99E-18	3.08E-16
173	ENSG00000136514	RTP4	151.7740378	1.430964502	0.25378953	1.72E-08	7.07E-07
174	ENSG00000095637	SORBS1	22.33209027	1.447812254	0.36473629	7.20E-05	0.0014124
175	ENSG00000119917	IFIT3	9346.640196	1.449047079	0.18515793	5.04E-15	4.92E-13
176	ENSG00000184979	USP18	322.0684426	1.467948376	0.20830342	1.83E-12	1.34E-10
177	ENSG00000174672	BRSK2	18.58883459	1.500165104	0.43605933	0.00058112	0.00819379
178	ENSG00000126709	IFI6	5742.083446	1.505819383	0.18215434	1.38E-16	1.69E-14
179	ENSG00000174469	CNTNAP2	91.76710651	1.521076879	0.17164444	7.87E-19	1.30E-16
180	ENSG00000185507	IRF7	401.0530617	1.52462822	0.12558695	6.48E-34	3.26E-31
181	ENSG00000132530	XAF1	2040.054972	1.553325181	0.19532048	1.82E-15	1.95E-13
182	ENSG00000188729	OSTN	29.63823625	1.571845852	0.32107575	9.80E-07	3.03E-05
183	ENSG00000119922	IFIT2	4971.873718	1.58279498	0.30960394	3.18E-07	1.11E-05
184	ENSG00000130303	BST2	515.5495804	1.606232166	0.26706427	1.81E-09	8.71E-08
185	ENSG00000135114	OASL	678.5997594	1.618086622	0.29500802	4.14E-08	1.63E-06
186	ENSG00000185745	IFIT1	9499.598383	1.625236973	0.27553788	3.67E-09	1.70E-07
187	ENSG00000144406	UNC80	33.35643479	1.645853163	0.28211885	5.41E-09	2.47E-07
188	ENSG00000157601	MX1	788.0643921	1.681938524	0.21314547	3.00E-15	3.02E-13
189	ENSG00000239713	APOBEC3G	8.18904619	1.691605569	0.55345102	0.00223961	0.02404933
190	ENSG00000137959	IFI44L	1895.516831	1.708381178	0.25640907	2.69E-11	1.69E-09
191	ENSG00000166960	CCDC178	7.26978729	1.752817694	0.63238705	0.00557561	0.04973312
192	ENSG00000111331	OAS3	1400.741081	1.764905695	0.19334849	6.97E-20	1.31E-17
193	ENSG00000226317	LINC00351	9.266894321	1.806647874	0.56683221	0.00143622	0.0171094
194	ENSG00000038945	MSR1	32.73363174	1.90576932	0.2834418	1.77E-11	1.15E-09
195	ENSG00000144278	GALNT13	6.60108041	1.919131542	0.66703098	0.00401317	0.03843387
196	ENSG00000089127	OAS1	1610.52137	1.921030489	0.42261977	5.48E-06	0.00014455
197	ENSG00000165949	IFI27	258.5776744	1.946606353	0.30782671	2.55E-10	1.43E-08
198	ENSG00000010030	ETV7	8.148691404	1.961426063	0.58560482	0.00080986	0.01076121
199	ENSG00000133083	DCLK1	22.7244345	2.032941519	0.34453054	3.62E-09	1.68E-07
200	ENSG00000185885	IFITM1	280.1140973	2.041462591	0.28144958	4.06E-13	3.23E-11
201	ENSG00000241106	HLA-DOB	5.471139168	2.128224123	0.76672129	0.00550751	0.0492893
202	ENSG00000090402	SI	42.51811075	2.204651064	0.25717636	1.01E-17	1.37E-15
203	ENSG00000133710	SPINK5	8.452237123	2.234851652	0.58886857	0.00014755	0.00259962
204	ENSG00000276231	PIK3R6	134.9643611	2.342026509	0.16248443	4.23E-47	4.55E-44
205	ENSG00000158560	DYNC1I1	6.377273324	2.424797676	0.72690522	0.0008506	0.01119177
206	ENSG00000182162	P2RY8	18.87220663	2.524684149	0.41364546	1.04E-09	5.33E-08
207	ENSG00000143110	C1orf162	11.78916585	2.544995348	0.52938005	1.53E-06	4.62E-05
208	ENSG00000186439	TRDN	25.75149955	2.609133191	0.37183102	2.27E-12	1.65E-10
209	ENSG00000100346	CACNA1I	9.6898529	2.653576793	0.70199317	0.00015679	0.00273739
210	ENSG00000256321	LOC101928441	5.44891664	2.944267937	0.83642378	0.00043144	0.00643285
211	ENSG00000115252	PDE1A	13.82895103	3.249650639	0.53806636	1.55E-09	7.57E-08
212	ENSG00000112562	SMOC2	7.057546302	3.816276727	0.8619899	9.54E-06	0.00023874
213	ENSG00000153064	BANK1	20.21074936	3.843776167	0.51467993	8.12E-14	7.08E-12
214	ENSG00000152503	TRIM36	868.8236345	4.302452142	0.09804759	0	0
215	ENSG00000139351	SYCP3	20.93493137	4.764018503	0.66082585	5.63E-13	4.38E-11
216	ENSG00000111729	CLEC4A	445.3379364	6.477572987	0.23627808	1.82E-165	1.46E-161

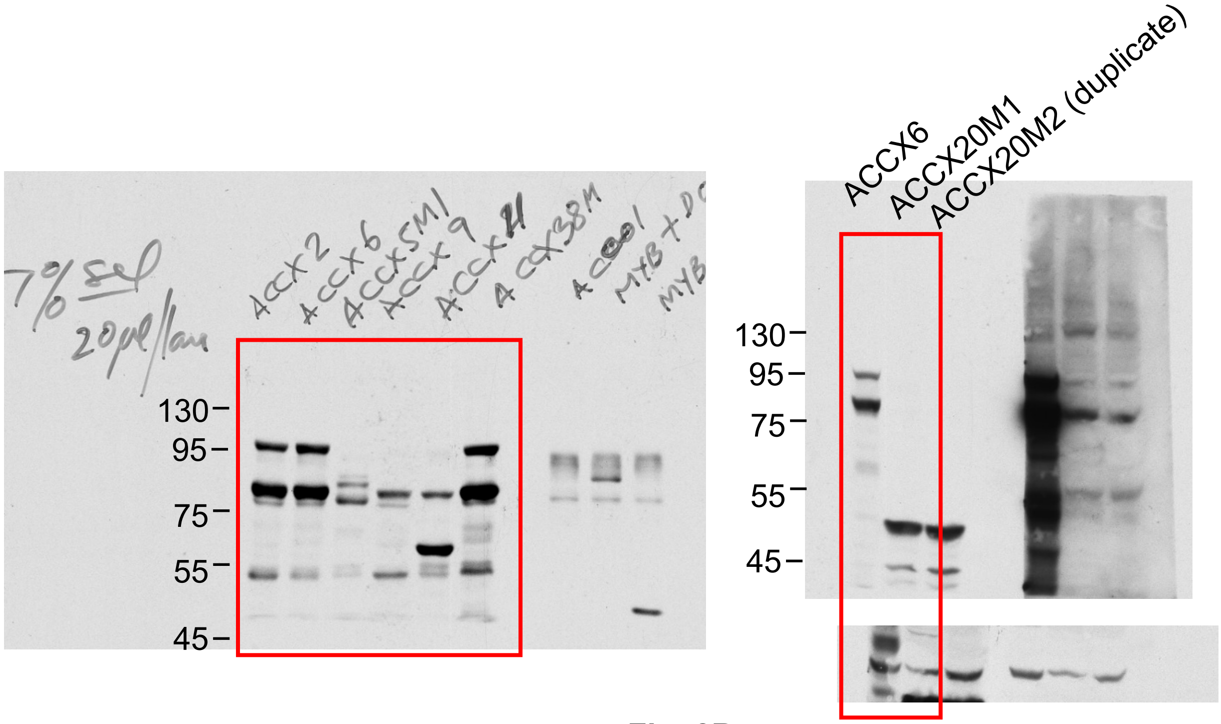

Fig. 2B

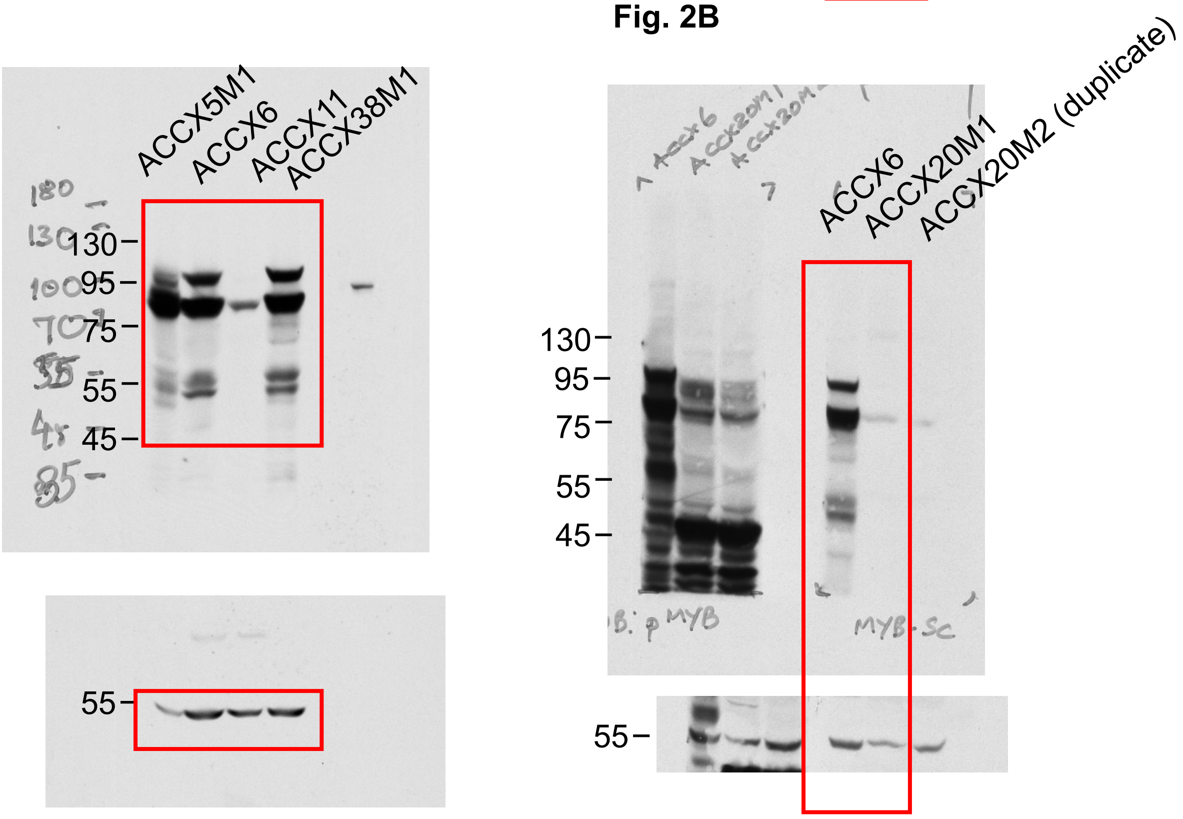

Fig 2C

Supplemental Fig. S1: Original Western blots for data shown in Fig 2. Red box indicates the cropped region.

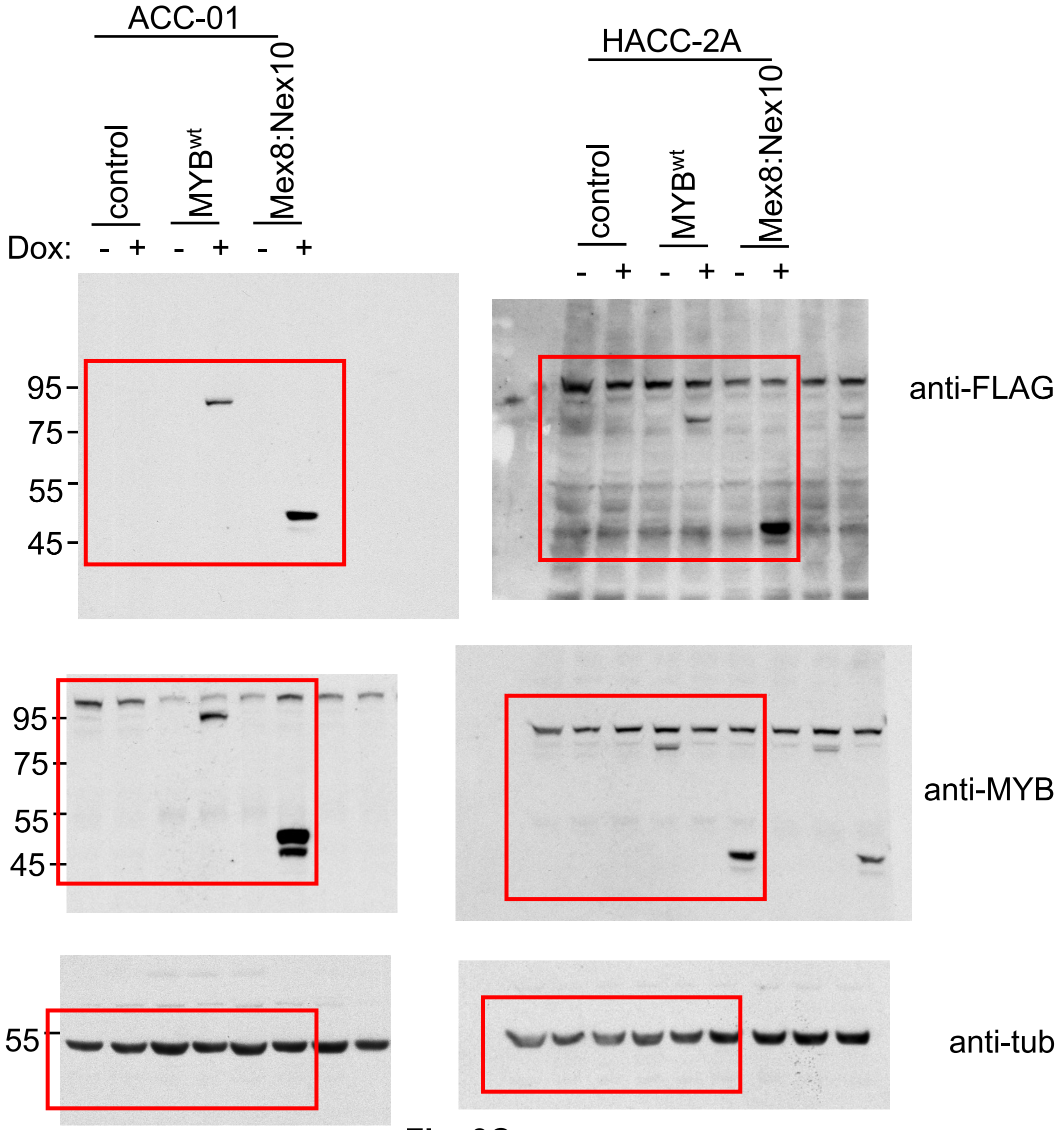

Fig. 3C

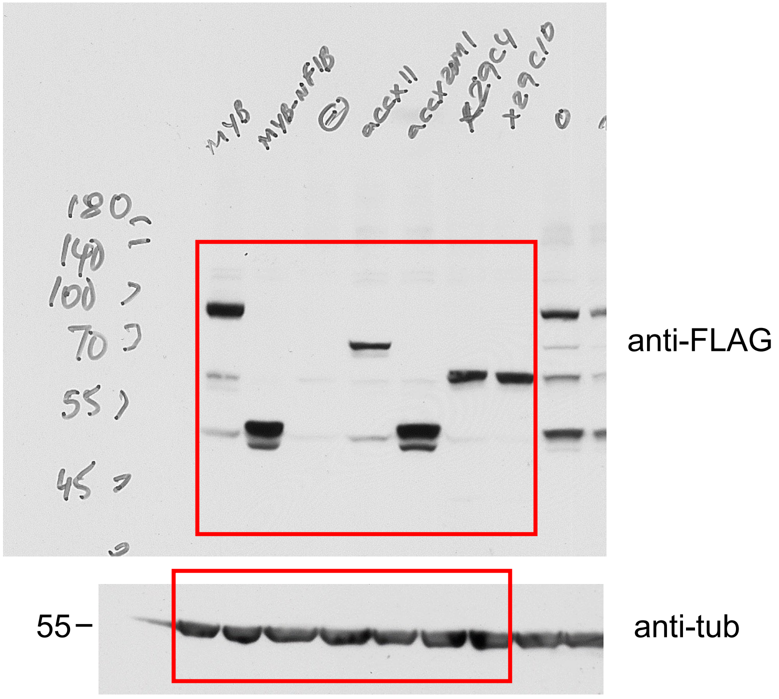

Fig. 3D

Supplemental Fig. S2: Original Western blots for data shown in Fig 3C and D. Red box indicates the cropped region.

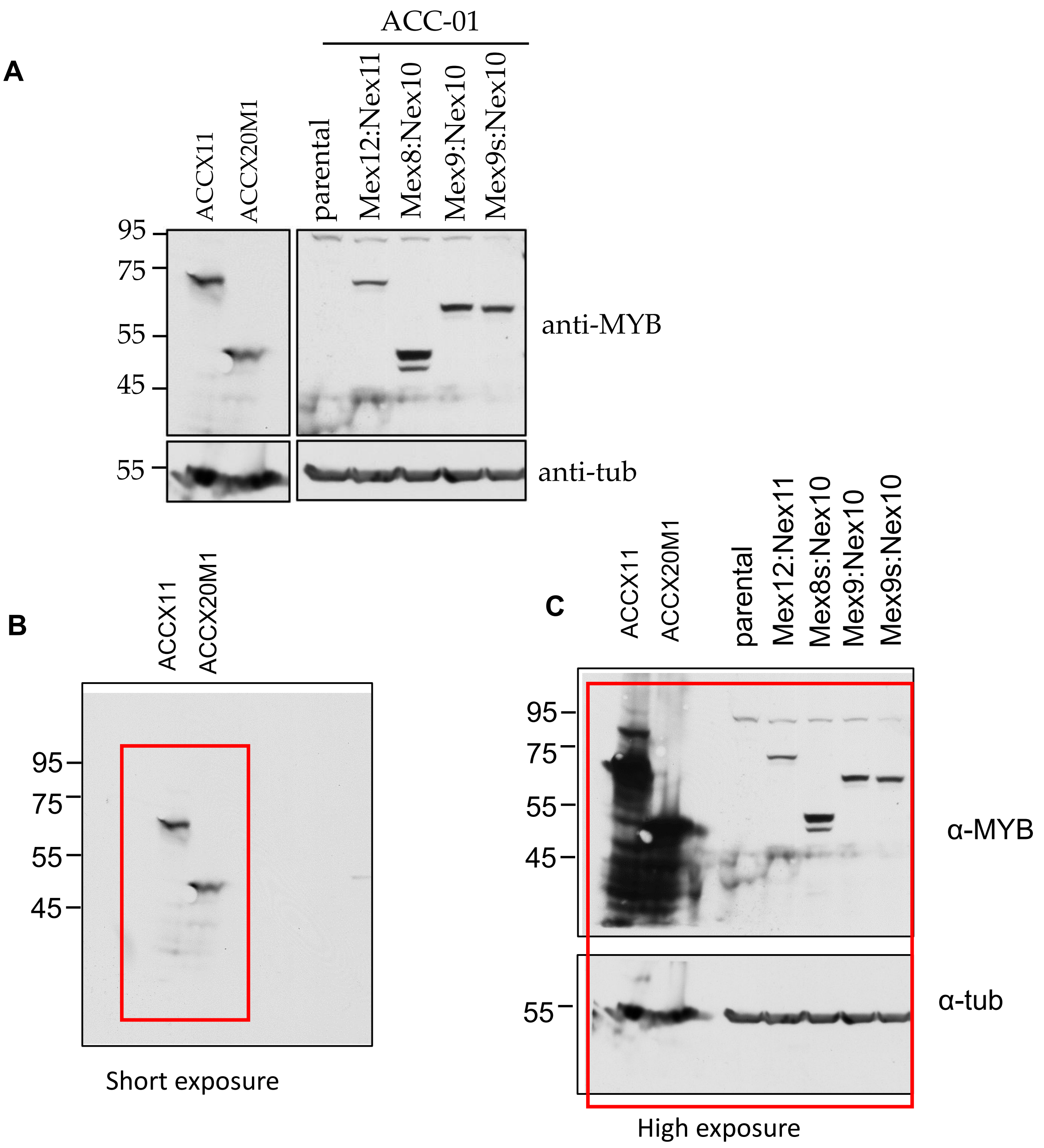

Supplemental Fig. S3: MYB-NFIB fusion expression in ACCX11 and ACCX20M1. (**A**), Cell lysates from the indicated patient-derived xenografts and ACC-01 cells expressing the various MYB-NFIB constructs were analyzed by immunoblotting using N-terminal epitope MYB antibody. Note that Mex12:Nex11 and Mex8:Nex10 were derived from ACCX11 and ACCX20M1, respectively. Although, there are no specific antibody that selectively detect MYB-NFIB fusion, this data supports the detectable MYB-NFIB fusion protein expression in ACCX11 and ACCX20M1. (**B** and **C**), Original Western blots of "**A**" with short and high exposed x-ray film.

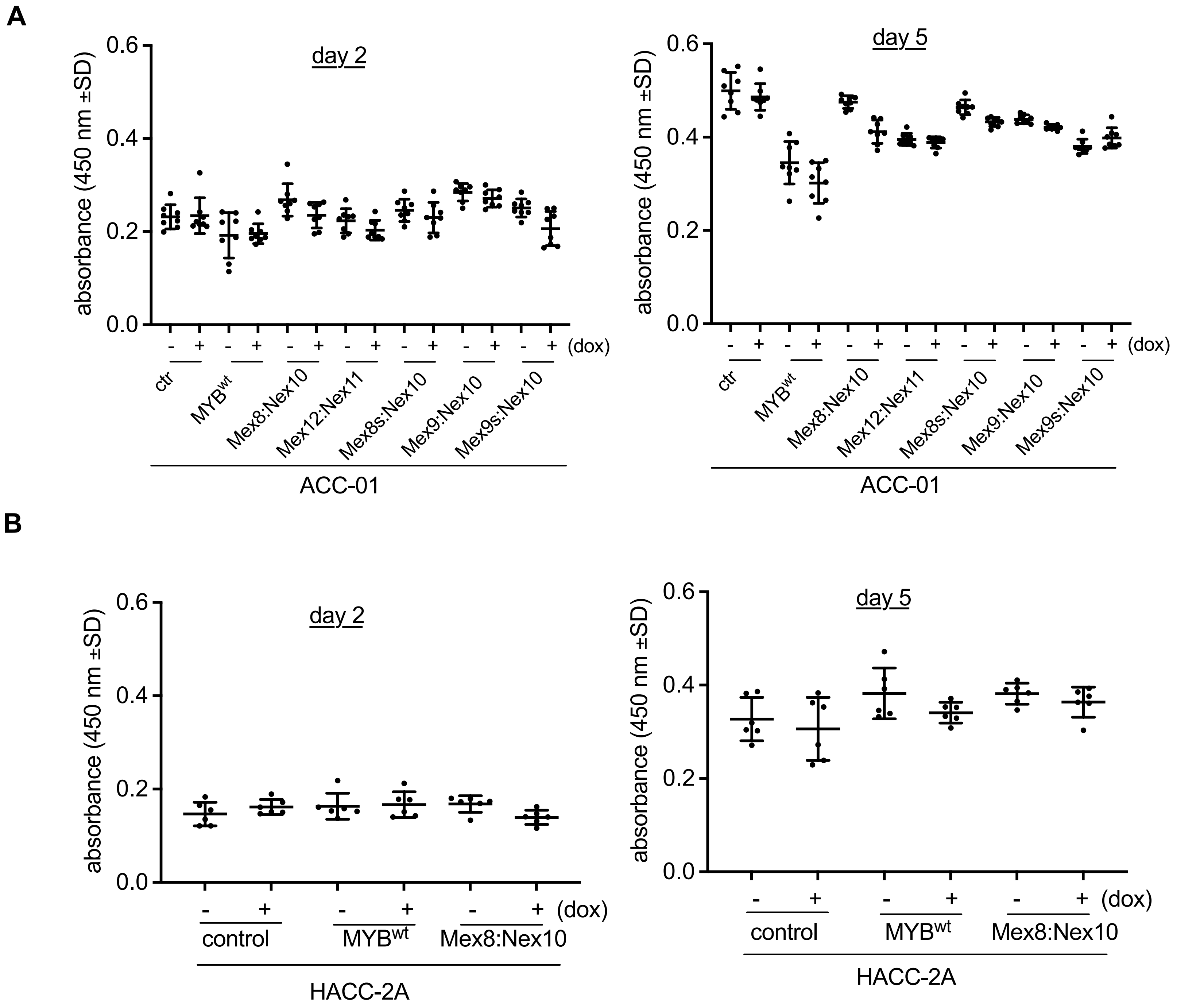

Supplemental Fig. S4: In vitro growth activities of MYB-MNFIB fusion in ACC cells. Indicated ACC cells were incubated either in the absence or presence of dox for up to 5 days. Change in growth activities was then measured by MTT assay. Note that expression of MYB or MYB-NFIB fusion does not impact cell growth activities in either ACC-01 (A) or HACC-2A cells.

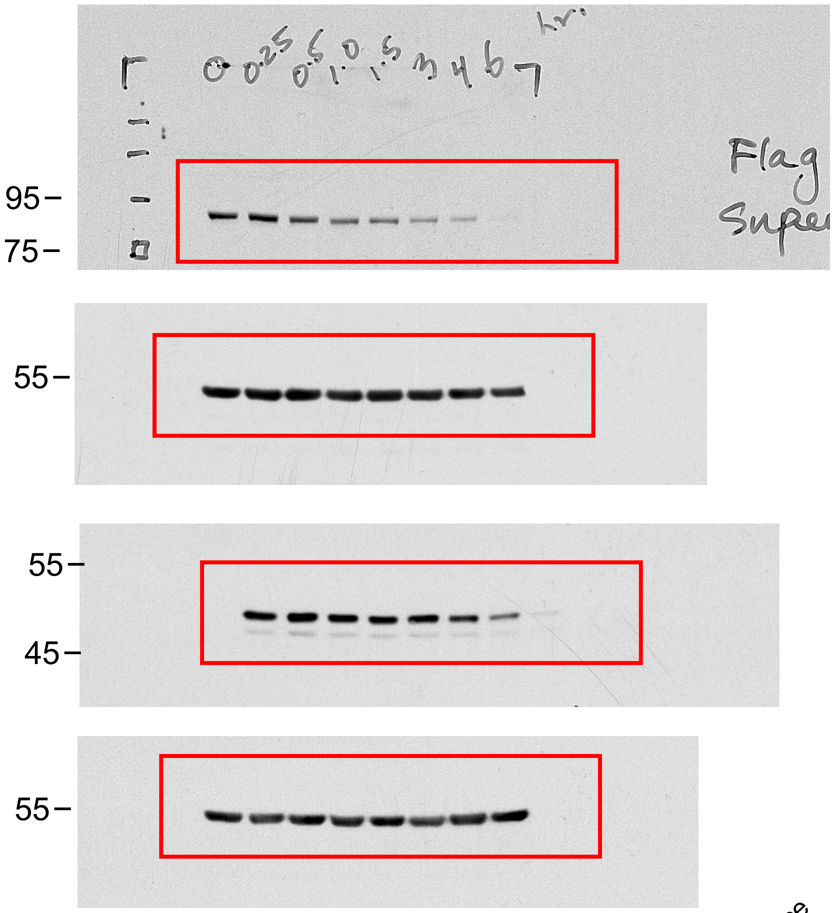

Fig. 4A

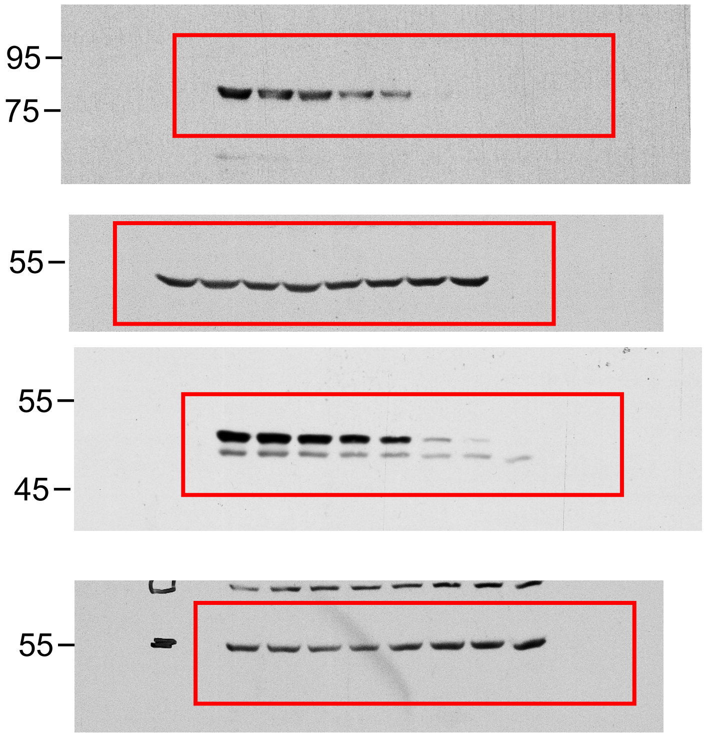

Fig 4C

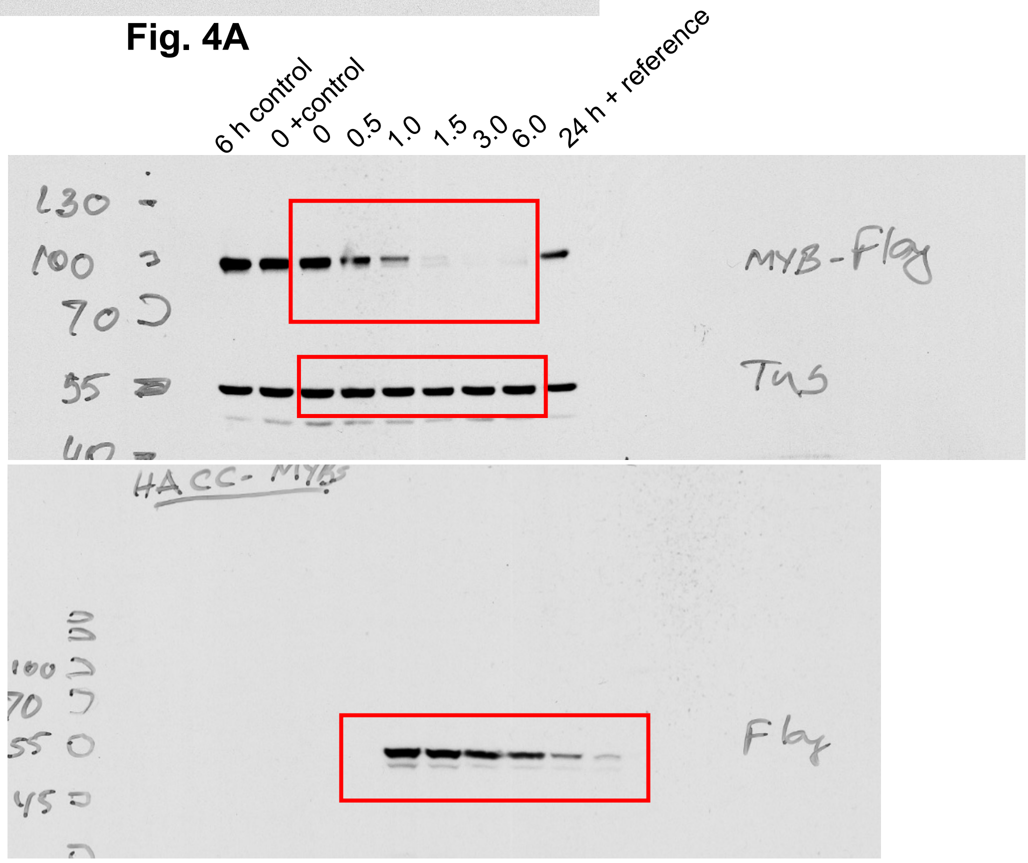

Fig. 4E

Supplemental Fig. S5: Original Western blots for data shown in Fig 4A, C and E. Red box indicates the cropped region.

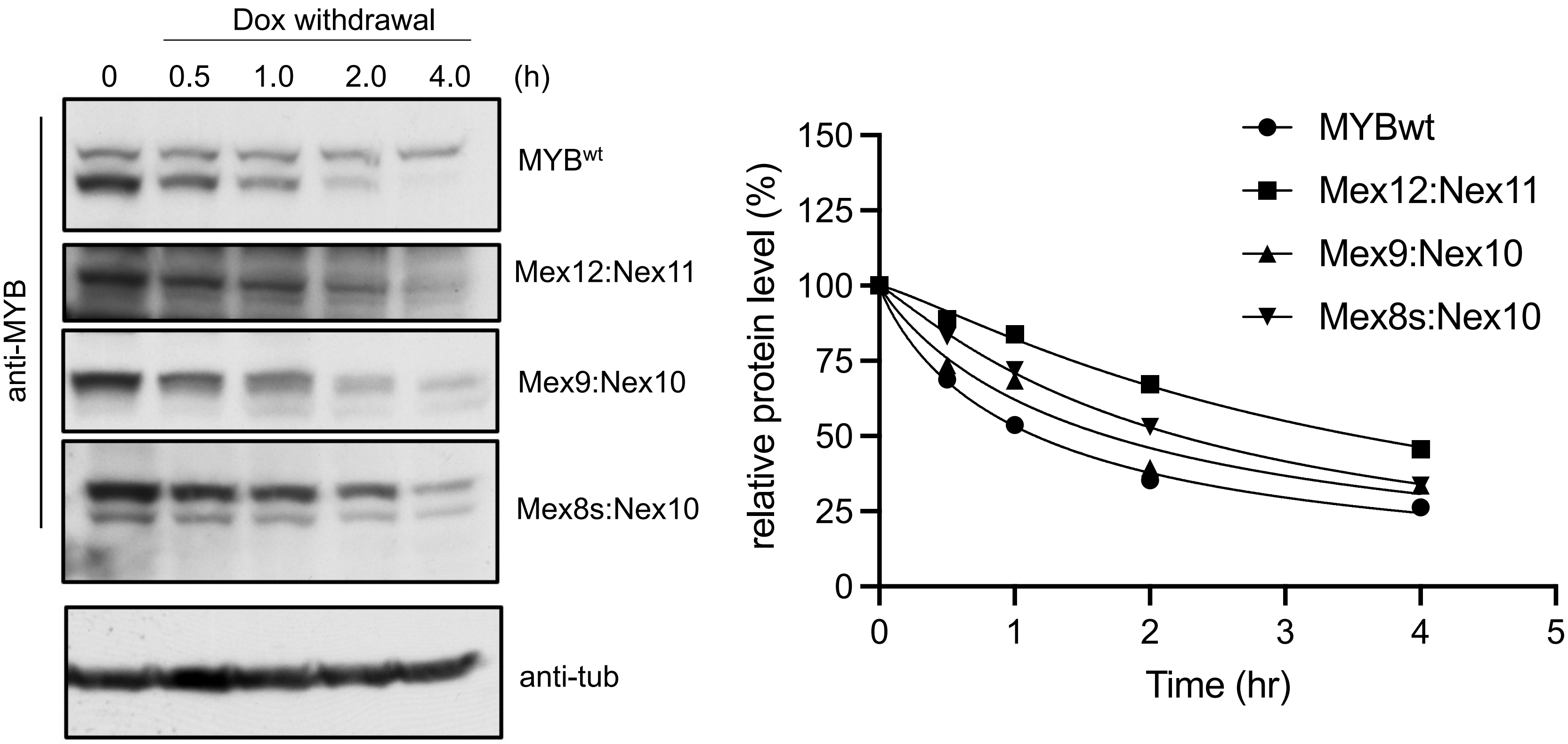

Supplemental Fig. S6. Differential protein stability of MYB^{wt} and MYB-NFIB fusion. (A), Stable ACC-01 cells harboring FLAG-tagged MYB^{wt} or variants of MYB-NFIB fusion were dox-induced for 24 h, then re-cultured in media without dox for the indicated time period. Cells lysates was prepared and analyzed by Western blotting using MYB antibody followed by densitometry to determine protein degradation pattern of MYB^{wt} and MYB-NFIB fusions.

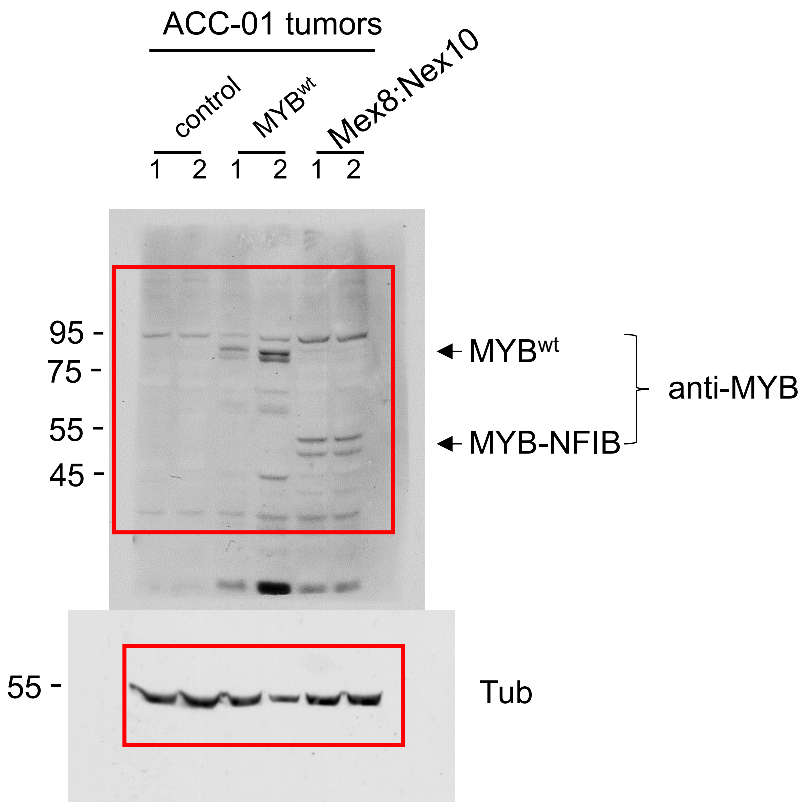

Fig. 6B

Supplemental Fig. S7: Original Western blots for data shown in Fig 6B. Red box indicates the cropped region.
